# Racial Health Equity and Social Needs Interventions: A Review of a Scoping Review

**DOI:** 10.1001/jamanetworkopen.2022.50654

**Published:** 2023-01-19

**Authors:** Crystal W. Cené, Meera Viswanathan, Caroline M. Fichtenberg, Nila A. Sathe, Sara M. Kennedy, Laura M. Gottlieb, Yuri Cartier, Monica E. Peek

**Affiliations:** 1 Department of Medicine, University of California, San Diego Health, San Diego; 2 School of Medicine, University of California, San Diego; 3 RTI International–University of North Carolina at Chapel Hill Evidence-based Practice Center, RTI International, Research Triangle Park; 4 University of California, San Francisco Social Intervention Research and Evaluation Network, San Francisco; 5 School of Medicine, Department of Family and Community Medicine, Center for Health and Community, University of California, San Francisco; 6 Section of General Internal Medicine, MacLean Center for Clinical Medical Ethics, Center for the Study of Race, Politics and Culture, The University of Chicago, Chicago, Illinois

## Abstract

**Importance:**

Social needs interventions aim to improve health outcomes and mitigate inequities by addressing health-related social needs, such as lack of transportation or food insecurity. However, it is not clear whether these studies are reducing racial or ethnic inequities.

**Objective:**

To understand how studies of interventions addressing social needs among multiracial or multiethnic populations conceptualize and analyze differential intervention outcomes by race or ethnicity.

**Evidence Review:**

Sources included a scoping review of systematic searches of PubMed and the Cochrane Library from January 1, 1995, through November 29, 2021, expert suggestions, and hand searches of key citations. Eligible studies evaluated interventions addressing social needs; reported behavioral, health, or utilization outcomes or harms; and were conducted in multiracial or multiethnic populations. Two reviewers independently assessed titles, abstracts, and full text for inclusion. The team developed a framework to assess whether the study was “conceptually thoughtful” for understanding root causes of racial health inequities (ie, noted that race or ethnicity are markers of exposure to racism) and whether analyses were “analytically informative” for advancing racial health equity research (ie, examined differential intervention impacts by race or ethnicity).

**Findings:**

Of 152 studies conducted in multiracial or multiethnic populations, 44 studies included race or ethnicity in their analyses; of these, only 4 (9%) were conceptually thoughtful. Twenty-one studies (14%) were analytically informative. Seven of 21 analytically informative studies reported differences in outcomes by race or ethnicity, whereas 14 found no differences. Among the 7 that found differential outcomes, 4 found the interventions were associated with improved outcomes for minoritized racial or ethnic populations or reduced inequities between minoritized and White populations. No studies were powered to detect differences.

**Conclusions and Relevance:**

In this review of a scoping review, studies of social needs interventions in multiracial or multiethnic populations were rarely conceptually thoughtful for understanding root causes of racial health inequities and infrequently conducted informative analyses on intervention effectiveness by race or ethnicity. Future work should use a theoretically sound conceptualization of how race (as a proxy for racism) affects social drivers of health and use this understanding to ensure social needs interventions benefit minoritized racial and ethnic groups facing social and structural barriers to health.

## Key Points

**Question:**

To what extent do studies of social needs interventions explain how race and ethnicity are conceptualized and used in analyses of intervention outcomes?

**Findings:**

Of the 152 studies conducted in multiracial or multiethnic populations within this review of a scoping review, 44 studies included race or ethnicity in their analyses, but these analyses were informative in only 21 studies (14%). Only 4 (9%) were conceptually thoughtful about what race or ethnicity means.

**Meaning:**

Social needs interventions have a unique opportunity to advance racial health equity if more attention is focused on conceptualization and use of race in intervention design and analysis.

## Teaser

This review of a scoping review examines how studies of interventions addressing social needs among multiracial or multiethnic populations conceptualize and analyze differential intervention outcomes by race or ethnicity.

## Introduction

Over the last decade, achieving health equity has been heralded as a key priority for health care delivery organizations. Health equity is achieved when all individuals have the opportunity to achieve their full health potential and no one is prevented from doing so.^1^ Achieving health equity requires addressing root causes of health inequities, including inequities in social and structural drivers (determinants) of health. Structural inequities (ie, differential access to goods, services, opportunities, and risks due to historical and current policies and practices) result in differential exposure to food insecurity, housing instability, and other drivers of poor health among groups based on social categorizations and identities (eg, race, ethnicity, gender, sexual orientation, and immigration status).

Understanding theoretical and conceptual underpinnings of race as a proxy for structural racism is critical for designing interventions that target root causes of health inequities. For example, an investigator may be interested in understanding contributors to higher stroke mortality among Black people compared with White people. An approach to evaluating this racial and ethnic inequity that is not conceptually thoughtful might singularly focus on individual-level behaviors or risk factors (eg, higher-fat diets, tobacco use, and hypertension) as opposed to examining the systems, policies, and practices that constrain or enable health behaviors and place individuals at risk of poorer outcomes. In reality, excess stroke risk is likely attributable to overrepresentation of Black people in underresourced communities with less access to both health-promoting and acute care resources, including comprehensive stroke centers. In this example, race is a proxy for neighborhood disadvantage. However, failure to provide this conceptual explanation has several detrimental consequences. First, it leaves the impression that there is something inherent or biological about minoritized racial or ethnic individuals that places them at higher risk of dying from stroke. Second, it may place responsibility on those individuals, instead of on the systems and structures that result in some neighborhoods having fewer resources and thereby more disadvantage than other neighborhoods. Further, this failure impedes our ability to identify actionable system-level, as opposed to individual-level, solutions.

Recently, efforts to develop and evaluate health care–based interventions to address unmet social needs have increased. Social needs are individual-level expressions of population-level drivers of health. Social needs interventions aim to improve health outcomes and mitigate health inequities by addressing material (eg, food and housing) and social (eg, physical safety) needs that are required for good health. For example, food insecurity has been associated with worse diabetes outcomes.^2^ Adults exposed to community violence have higher odds of elevated blood pressure.^2^ Because of historical and ongoing structural racism, unmet social needs are more prevalent among minoritized racial and ethnic populations.

Minoritized racial and ethnic groups also experience socioeconomic disadvantage differently than White people. For example, because of redlining and other forms of institutional and interpersonal racism, Black families experiencing poverty typically live in neighborhoods with higher concentrations of poverty, worse-quality housing and schools, and fewer community resources than White families with the same income.^3,4,5,6^ Consequently, social needs interventions to improve housing stability or food insecurity may be less accessible to or effective for Black individuals. In addition, minoritized racial and ethnic groups face greater barriers, including interpersonal racism and discrimination, to accessing services and resources to help mitigate unmet social needs. Finally, social needs interventions could be less effective in minoritized racial and ethnic populations because of low self-efficacy resulting from internalized racism. Despite many ways racism may alter the effectiveness of social needs interventions, to our knowledge, no one has yet examined the extent to which social needs intervention studies have explicitly considered whether and how minoritization based on race or ethnicity might affect intervention effectiveness.

To fill these knowledge gaps, we built on the Patient-Centered Outcomes Research Institute’s (PCORI’s) recent scoping review and evidence map of social needs interventions in health care settings^7^ to explore how these studies conceptualize and analyze differential intervention outcomes by race or ethnicity.

## Methods

### Scope of the Review

This synthesis was conducted as a “rapid review,” which is defined as a form of knowledge synthesis that accelerates the process of conducting a traditional systematic review through streamlining or omitting specific methods to produce evidence for stakeholders in a resource-efficient manner,^8^ but for which a reporting guideline has not yet been released. With this type of review, specific methodological adjustments were planned: (1) reliance on existing searches for the evidence map; (2) no second review of risk of bias (that is, we relied on the evidence map approach of single risk-of-bias ratings with spot checks); (3) single reviewer recheck of data for subgroup or effect modification analyses; (4) focused data extraction outcomes; (5) no strength of evidence grading; and (6) a primarily narrative or qualitative synthesis.

For this review, we focused on studies in multiracial or multiethnic populations to facilitate our ability to examine differential intervention outcomes by race or ethnicity. We addressed the following key questions:

1. How many studies include race or ethnicity in their analyses? Among those that do, what social needs have been addressed and what interventions have been studied?
2. Among studies that include race or ethnicity in their analyses, how do they conceptualize race or ethnicity?
3. How many studies examine whether intervention effects differ based on the race or ethnicity of participants? Among studies that do, how do impacts vary?
4. What is the overlap between studies addressing the conceptualization of race or ethnicity (thoughtfulness) and use of race or ethnicity to examine differential impact (informativeness)?

### Data Sources and Searches

This review was based on a PCORI-funded scoping review and evidence map of social needs interventions in health care settings.^7^ The PCORI review included searches of MEDLINE and the Cochrane Library conducted between January 1, 1995, and November 29, 2021, as well as references of relevant systematic reviews, companion articles, and consultation with subject matter experts (eMethods and eTables 1-7 in Supplement 1). We registered the protocol in the Open Science Framework (September 17, 2021)^9^ and adhered to guidelines from the Preferred Reporting Items for Systematic Reviews and Meta-analyses (PRISMA) reporting guideline and the PRISMA extension on equity.^10,11,12^ This review and synthesis was conducted between December 2021 and November 2022.

### Study Selection

In Supplement 1, eTable 8 and eFigure 1 detail the criteria used to select studies for PCORI’s scoping review and evidence map.^7^ Briefly, that review selected English-language studies set in the US that addressed individual social needs (as defined by Healthy People 2020 and Healthy People 2030).^13,14^ We required that studies report at least 1 of the following outcomes: behavioral outcomes, health outcomes, health care utilization outcomes, and harms or unanticipated outcomes. For this review, we further modified inclusion criteria to focus on studies with 2 or more racial or ethnic groups. Two investigators (S.M.K., N.A.S., M.V., and/or other authors of the PCORI evidence map^7^) independently reviewed titles, abstracts, and full-text articles; disagreements were resolved by discussion or by a third reviewer (S.M.K., N.A.S., M.V., and/or other authors of the PCORI evidence map^7^).

### Data Extraction and Quality Assessment

For the PCORI scoping review and evidence map, we extracted population and intervention characteristics, social needs addressed, recruitment setting, intervention setting, and intervention provider. For this review, we also extracted racial or ethnic composition of the study sample, including how race or ethnicity was conceptualized; whether and how race or ethnicity variables were included in analyses; and specific outcomes reported by race or ethnicity. For each included study, 1 reviewer extracted relevant study characteristics and outcomes, and a second reviewer checked data for completeness and accuracy (M.V., N.A.S., S.M.K., and/or other authors of the PCORI evidence map^7^); 1 reviewer (M.V.) assessed risk of bias of included studies, and a second nonauthor reviewer spot-checked the studies (eAppendix 2, eTables 9 and 10 in Supplement 1).

### Data Synthesis and Analysis

**Figure 1.**
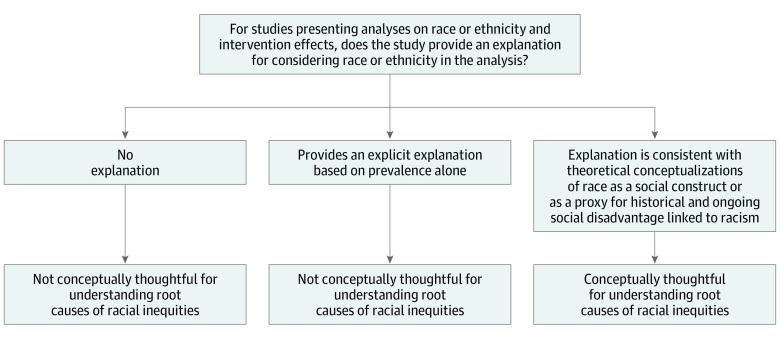
Identifying Social Needs Intervention Studies That Are Conceptually Thoughtful This figure outlines a process for assessing the conceptual thoughtfulness for understanding root causes of racial health inequities of social needs interventions studies with multiple racial or ethnic groups.

**Figure 2.**
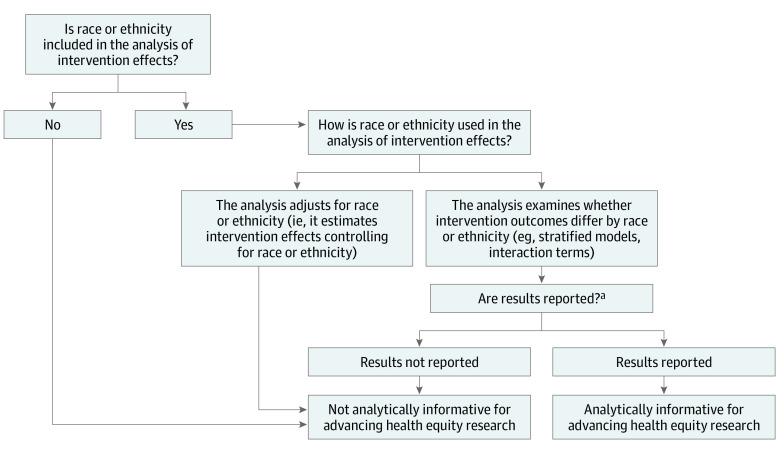
Identifying Social Needs Intervention Studies That Are Analytically Informative for Advancing Racial Health Equity Research This figure outlines a process for assessing analytical informativeness for advancing racial health equity of studies of social needs interventions. These studies examine whether intervention effects differ by race or ethnicity. ^a^Results could be reported in brief (eg, as a statement of no differences), in detail, in the main report, or in supplemental material.

To answer our key questions, we assessed whether studies included race or ethnicity variables in analyses of intervention effects, and we described those studies. Among those that did include race or ethnicity in their analyses, we examined how race or ethnicity was conceptualized. Specifically, we assessed (1) if there was any explanation given for the use of race or ethnicity in the analyses and (2) whether the explanation, if provided, was consistent with current understanding of race as a social construct and proxy for various forms of racialized disadvantage (eg, neighborhood disadvantage, structural racism, implicit bias). We considered studies that explicitly provided such explanations for race to be conceptually thoughtful for understanding root causes of racial health inequities (Figure 1). We also determined whether studies tested for differential intervention effects by race or ethnicity, either by stratifying analyses by race or ethnicity or by including interaction terms (also known as effect modification) (Figure 2). Studies that examined and reported differential intervention effects by race or ethnicity were labeled analytically informative for advancing racial health equity research.

These 2 sets of analyses generated a framework that categorized studies on whether they were conceptually thoughtful and analytically informative for advancing racial health equity research. We developed this framework after reviewing multiple critiques of the current approach to conducting and reporting research to advance racial health equity,^15,16,17,18,19,20^ and we simplified the critiques into what we perceived to be the fundamental concerns: conceptual and methodological issues.

## Results

Among the 157 studies identified by the PCORI scoping review, 152 were among multiracial or multiethnic populations. These studies met inclusion criteria for this review^7^ (eAppendix 1 and eFigure 2 in Supplement 1).

### Number and Characteristics of Studies Including Race and Ethnicity in their Analyses

Among 152 studies in multiracial or multiethnic populations,^21,22,23,24,25,26,27,28,29,30,31,32,33,34,35,36,37,38,39,40,41,42,43,44,45,46,47,48,49,50,51,52,53,54,55,56,57,58,59,60,61,62,63,64,65,66,67,68,69,70,71,72,73,74,75,76,77,78,79,80,81,82,83,84,85,86,87,88,89,90,91,92,93,94,95,96,97,98,99,100,101,102,103,104,105,106,107,108,109,110,111,112,113,114,115,116,117,118,119,120,121,122,123,124,125,126,127,128,129,130,131,132,133,134,135,136,137,138,139,140,141,142,143,144,145,146,147,148,149,150,151,152,153,154,155,156,157,158,159,160,161,162,163,164,165,166,167,168,169,170,171,172^ 44 studies^23,26,28,29,30,34,35,47,58,62,63,66,68,74,78,80,82,83,85,87,92,93,94,95,96,101,102,117,126,128,129,135,142,143,151,159,161,163,165,167,168,169,170,171^ (28%; comprising 49 interventions) included race or ethnicity variables in their analyses in some way. eTable 11 in Supplement 1 outlines the key characteristics of these 44 studies and 49 interventions. The interventions most commonly targeted the following social needs: health care services access and quality (n = 30), housing stability and quality (n = 19), transportation assistance (n = 15), and food insecurity (n = 14).

#### Conceptualization of Race or Ethnicity

Among 44 studies that included race or ethnicity in their analyses, only 4 (9%) were categorized as conceptually thoughtful for understanding root causes of racial health inequities (eTables 12 and 13 in Supplement 1).^28,142,161,165^ In other words, only 4 studies explicitly or implicitly noted that race or ethnicity are markers of exposure to racism. Towfighi et al^161^ noted that Black and Latino communities are disproportionately underresourced and experience disparities in access to quality health care. Krieger et al^28^ attributed part of the increased risk of asthma morbidity among low-income, minoritized racial groups to substandard housing. Szilagyi et al^165^ described complex and multifactorial reasons (individual, physician, health system access barriers, and cost) for an immunization gap between White and Black or Hispanic children, and Crisanti et al^142^ noted that structural racism may account for poorer outcomes in minoritized participants. None of the 4 conceptually thoughtful studies provided the conceptualization of race or ethnicity in the introduction or methods sections, where one may expect to find such explanations if they are helping to frame the manuscript or guide analyses; instead, explanations were in discussion sections, where they were used to help interpret study findings. Further, 2 of the 4 conceptually thoughtful studies included their conceptualization of race or ethnicity in companion publications rather than the main outcomes publication.

#### Examination of Differential Impacts of Interventions by Race or Ethnicity

Among 152 studies in multiracial or multiethnic populations, only 21 (14%)^26,28,29,47,62,68,78,80,87,93,95,96,101,126,128,151,159,161,165,170,171^ reported whether intervention outcomes differed by race or ethnicity of participants. Another 23 studies^23,30,34,35,58,63,66,74,82,83,85,92,94,102,117,129,135,142,143,163,167,168,169^ (15%) included race or ethnicity in their analyses as confounders. The rest (108 [71%]) did not include race or ethnicity in their analyses at all. Table 1 provides brief intervention characteristics and outcomes for the 21 studies that examined differential outcomes by race or ethnicity, categorized along the axes of conceptual thoughtfulness and analytical informativeness, and organized by category of intervention. Two-thirds of the studies (14 of 21 studies [67%])^28,62,68,78,87,93,95,96,101,126,151,161,170,171^ categorized as analytically informative reported no differences in intervention outcomes by race or ethnicity. Among the 7 studies that did find differential intervention outcomes by race or ethnicity,^26,29,47,80,128,159,165^ 6 were studies of relatively intense case management or community health worker/peer mentor outreach in diverse settings, and 1 addressed the Reach Out and Read–based intervention for children (Table 2).

**Table 1.** Racial Health Equity and Social Needs Interventions: Intervention Characteristics and Results in 21 Studies With Analytically Informative and Conceptually Thoughtful Analyses^a^

Source	Design	Quality	Participnats, No.	Tailored	Explores root causes of racial health inequities	Breakdown of race or ethnicity	Outcomes for overall population		
							Health	Behavioral	Utilization
**Conceptually thoughtful for understanding root causes of racial health inequities and analytically informative for advancing racial health equity research**									
**Improving access to health care or social services through care coordination or assistance using bridge personnel**									
Krieger et al,^28^ 2005	RCT	Low	274	Yes	Yes	No single group was a majority	Mixed	NA	Positive
						Caregiver ethnicity (%): High intensity: non-Hispanic White (12.3); non-Hispanic African American (31.9); Vietnamese (25.4); other Asian (9.4); Hispanic (17.4); other (3.6). Low intensity: non-Hispanic White (21.3); non-Hispanic African American (27.9); Vietnamese (22.1); other Asian (5.2); Hispanic (17.7); other (5.9)			
Szilagyi et al,^165^ 2002	Single group^b^	NR	10 066	Yes	Yes	Majority varies by site	NA	NA	Positive
						Inner city, %: Black (non-Hispanic): 58; Hispanic: 21; White (non-Hispanic): 15; Asian and others: 6			
						Rest of city, %: Black (non-Hispanic): 37; Hispanic: 15; White (non-Hispanic): 38; Asian and others: 10			
						Suburbs, %: Black (non-Hispanic): 7; Hispanic: 3; White (non-Hispanic): 84; Asian and others: 6			
						County, %: Black (non-Hispanic): 28; Hispanic: 10; White (non-Hispanic): 55; Asian and others: 7			
Towfighi et al,^161^ 2021	RCT	High	487	Yes	Yes	Majority White/non-Hispanic White	Mixed	Mixed	Mixed
						Overall, No. (%): White: 335 (70.4); Black: 87 (18.3); Asian: 30 (6.3)			
						≥1 Race, No. (%): 10 (2.1); Native American or Alaska Native: 9 (1.9); Native Hawaiian or other Pacific Islander: 5 (1.1); Hispanic ethnicity: 347 (71.3)			
**Not conceptually thoughtful for understanding root causes of racial health inequities but analytically informative for advancing racial health equity research**									
**Improving access to health care or social services through care coordination or assistance using bridge personnel**									
Duncan et al,^170^ 2020	RCT	High	5882	No	No	Majority White/non-Hispanic White	None	None	None
						Intervention, No. (%): White: 2112 (79.1); non-White: 559 (20.8); Missing: 18 (0.67)			
						Usual care, No. (%): White: 2122 (67.2); non-White: 1037 (32.5); Missing: 34 (1.1) (data for non-White calculated)			
Foster et al,^151^ 2018	NRS	Low	85 701	No	No	No single group was a majority	NA	NA	None
						Referred-successful linkage, No. (%): African American: 646 (61); Caucasian: 338 (31.9); other/not documented: 63 (5.9); Hispanic: 6 (0.6); Asian: 6 (0.6)			
						Referred-unsuccessful linkage, No. (%): African American: 403 (64.1); Caucasian: 187 (29.7); other/not documented: 33 (5.2); Hispanic: 5 (0.8); Asian: 1 (0.2)			
						Referred-assistance declined, No. (%): African American: 262 (57.7); Caucasian: 154 (33.9); other/not documented: 30 (6.6); Hispanic: 7 (1.5); Asian: 1 (0.2)			
						Nonreferred, No. (%): African American: 34 581 (41.3); Caucasian: 39 386 (47.1); other/not documented: 8061 (9.6); Hispanic: 1146 (1.4); Asian: 463 (0.6)			
Glendenning-Napoli et al,^80^ 2012	Single group^b^	NR	83	No	No	Majority White/non-Hispanic White	NA	NA	Positive
						No. (%): Non-Hispanic White: 43 (51.8); Hispanic: 19 (22.9); African American: 21 (25.3)			
Hilgeman et al,^128^ 2014	RCT	High	203	No	No	Majority White/non-Hispanic White	NA	NA	Positive
						Intervention, No. (%): White: 52 (51.49); Black: 49 (48.51); Asian: 0; Hispanic: 0			
						Comparison, No. (%): White: 67 (64.42); Black: 34 (62.69); Asian: 1 (0.96); Hispanic: 2 (1.92)			
Juillard et al,^26^ 2016	Single group^b^	NR	459	Yes	No	No single group was a majority	Positive	NA	NA
						No. (%): Black/African American: 215 (46.8); Latino: 200 (43.5); White: 23 (5.0); other (Native American, native Alaskan, native Hawaiian, Asian Pacific Islander, and mixed race): 21 (4.5)			
Kelley et al,^68^ 2020	RCT	High	100	Yes	No	No single group was a majority	NA	NA	Mixed
						Intervention, No. (%): White, non-Hispanic/Latino: 6 (12.24); Black, non-Hispanic/Latino: 23 (46.94); Hispanic/Latino: 19 (38.78); other: 1 (2.04)			
						Usual care, No. (%): White, non-Hispanic/Latino: 12 (23.53); Black, non-Hispanic/Latino: 25 (49.02); Hispanic/Latino: 14 (27.45); other: 0			
Krieger et al,^96^ 1999	RCT	Low	241	Yes	No	Majority Black/non-Hispanic Black	NA	NA	Positive
						Intervention (%): Black (79.4)			
						Control (%): Black (78.8)			
Krieger et al,^87^ 2009	RCT	Medium	309	Yes	No	No single group was a majority	Mixed	NA	None
						Enrolled in study (%): White (11.3); African American (20.1); Vietnamese (11.0); other Asian (5.8); Hispanic (47.9); other: (3.9)			
						Completed study (%): White (10.3); African American (20.3); Vietnamese (10.7); other Asian (5.5); Hispanic (49.8); other (3.3)			
Krieger et al,^95^ 2015	RCT	Medium	366	Yes	No	No single group was a majority	Mixed	Positive	None
						Intervention (%): White (26.0); Black (16.9); Hispanic (48.6); other (8.5)			
						Control (%): White (31.2); Black (16.4); Hispanic (45.0); other (7.4)			
Lapham et al,^101^ 1995	CE^c^	NR	469	Yes	No	No single group was a majority	NA	Mixed	NA
						Overall (%): Non-Hispanic White (41); Hispanic White (Hispanic) (31); Native American (18); other race groups (10)			
Lyles et al,^159^ 2021	Single group^b^	NR	618	Yes	No	Majority Black/non-Hispanic Black	Positive	NA	NA
						No. (%): Black: 318 (51); Hispanic/LatinX: 145 (23); White: 35 (6); Asian: 5 (1); other: 45 (7); missing/unknown: 70 (11)			
Slesnick et al,^62^ 2007	Single group^b^	NR	172	No	No	No single group was a majority	NA	Positive	Positive
						White (37.2%); Hispanic (31.4%); Native American (12.2%); African American or Black (7.6%); mixed ethnicity (11.6%)			
Tessaro et al,^47^ 1997	NRS	Low	14 714	No	No	Majority Black/non-Hispanic Black	None	NA	Mixed
						Maternal outreach worker program (%): African American (61.8); Caucasian (38.2)			
						Care coordination program (%): African American (59.4); Caucasian (40.6)			
Xiang et al,^78^ 2019	Single group^b^	NR	586	No	No	Majority Black/non-Hispanic Black	Mixed	Mixed	Mixed
						White (39.8%); African American (52.7%); other (7.5%)			
**Improving access to health care or social services through referrals, no care coordination or bridge personnel**									
Chan et al,^126^ 2009	Single group^b^	NR	725	No	No	NR	NA	NA	Positive
**Transportation assistance**									
Whorms et al,^171^ 2021	Single group^b^	NR	15 577	No	No	Majority White/non-Hispanic White	NA	NA	Mixed
						Rideshare appointments, No.: White: 114; Black/African American: 11; Asian: 8; Hispanic: 12; other: 3			
						Nonrideshare appointments, preintervention, No.: White: 6041; Black/African American: 383; Asian: 357; Hispanic: 749; other: 491			
						Nonrideshare appointments, postintervention, No.: White: 5769; Black/African American: 353; Asian: 277; Hispanic: 720; other: 215			
Chaiyachati et al,^93^ 2018	NRS	Medium	786	No	No	Majority Black/non-Hispanic Black	NA	NA	Mixed
						Intervention, No. (%): White: 10 (2.5); Black: 371 (94.2); other/mixed: 13 (3.3); Hispanic: 2 (0.5); non-Hispanic: 392 (99.5)			
						Control, No. (%): White: 4 (1.0); Black: 377 (96.2); other/mixed: 11 (2.8); Hispanic: 1 (0.3); non-Hispanic: 391 (99.7)			
**Early childhood development and education**									
Mendelsohn et al,^29^ 2001	NRS	Medium	138	No	No	Majority Hispanic/Latino	NA	Mixed	NA
						Intervention (families, %): Latino: 79.6%; Black: 20.4%			
						Comparison (families, %): Latino: 64.4%; Black: 35.6%			

Abbreviations: CE, comparative effectiveness; NA, not applicable; NR, not reported; NRS, nonrandomized study with comparison arms, includes experimental and observational designs; RCT, randomized controlled trial.

^a^ Social needs interventions often include multiple components and could be characterized in multiple ways. In this table, key intervention-specific features were used to characterize studies rather than population-specific features (eg, peer counseling and support in participants experiencing homelessness were characterized as “improving access to health care or social services care coordination or assistance using bridge personnel” rather than offering housing support). eTables 11 and 12 in Supplement 1 list detailed intervention characteristics and social needs addressed. Bridge personnel include community health workers, peer mentors, and health navigators.

^b^ Preintervention to postintervention changes or changes over time serve as the proxy for the intervention effect in single-arm studies.

^c^ Each group in CE studies was treated as a single-arm design to understand the intervention’s outcomes over time.

**Table 2.** Contribution of Race or Ethnicity Analyses to Understanding Impacts of Intervention on Racial Health Equity in 7 Studies Reporting Differential Effects

Source	Design	Quality	Participants, No.	Contribution of race or ethnicity analyses to understanding impacts of intervention on racial health equity
**Conceptually thoughtful for understanding root causes of racial health inequities and analytically informative for advancing racial health equity research**				
Szilagyi et al,^165^ 2002	Single group^a^	NR	10 066	Disparities in White-Black and White-Hispanic immunization rates declined over time
**Not conceptually thoughtful for understanding root causes of racial health inequities but analytically informative for advancing racial health equity research**				
Glendenning-Napoli et al,^80^ 2012	Single group^a^	NR	83	Significant pre-post declines in acute outpatient encounters in Hispanic and African American participants but not non-Hispanic White participants Significant pre-post declines in inpatient admission and increases in clinic visits for all 3 race or ethnicity groups
Hilgeman et al,^128^ 2014	RCT	High	203	No significant interactions between race and intervention groups and clinic attendance Black veterans in control group took longer to attend appointment than White veterans; no differences by race in the intervention group
Juillard et al,^26^ 2016	Single group^a^	NR	459	Significantly lower rates of reinjury over time among minoritized (Black, Latino, other) populations vs White population No significant differences by race or ethnicity in whether the intervention met client needs
Lyles et al,^159^ 2021	Single group^a^	NR	618	Improvement in mean HbA_1c_ among Black and Hispanic/Latinx participants slightly larger than among White participants; statistical significance not assessed
Tessaro et al,^47^ 1997	NRS	Low	14 714	Lower rate of observed vs expected low/very low birth weight among African American participants; no differences for White participants Less adequate prenatal care among African American participants than control participants; no differences by intervention group for Caucasian participants
Mendelsohn et al,^29^ 2001	NRS	Med	138	Significantly better vocabulary scores in Latino families receiving intervention

Abbreviations: HbA_1c_, hemoglobin A_1c_; Med, medium; NR, not rated; NRS, nonrandomized study; RCT, randomized controlled trial.

^a^ Preintervention to postintervention changes or changes over time serve as the proxy for the intervention outcome in single-arm studies.

Among the 7 studies that reported differential intervention outcomes, 4 found that the interventions benefited minoritized racial or ethnic populations more than White populations or reduced inequities in minoritized compared with White populations.^26,128,159,165^ Among the 3 remaining studies, 1 reported better outcomes in Latino children receiving the intervention when compared with those not receiving the intervention.^29^ In that study, however, there was not a statistically significant difference between intervention and comparison clinics, which also included Black participants. The 2 remaining studies^47,80^ found mixed health equity outcomes: for some outcomes, minoritized racial or ethnic participants benefited more, and for other outcomes, White participants benefited more.

#### Conceptually Thoughtful and Analytically Informative Studies

When we considered the combination of conceptual thoughtfulness and analytical informativeness among studies that included race or ethnicity variables in their analyses, half of the studies (22 [50%]) were considered neither conceptually thoughtful for understanding root causes of racial health inequities nor analytically informative for advancing racial health equity research (Table 3).^23,30,34,35,58,63,66,74,82,83,85,92,94,102,117,129,135,143,163,167,168,169^ More than one-third (18 [41%]) were characterized as analytically informative but not conceptually thoughtful.^26,29,47,62,68,78,80,87,93,95,96,101,126,128,151,159,170,171^ Among the 21 analytically informative studies, only 3 were also categorized as conceptually thoughtful.^28,161,165^ One study (2%)^142^ was conceptually thoughtful but not analytically informative: thoughtful because the authors attributed racial and ethnic differences in 1 of the outcomes—psychological distress—to structural racism, but noninformative because analyses of intervention outcomes were adjusted for race or ethnicity rather than stratifying or testing for outcome modification by race or ethnicity.

**Table 3.** Categorization of Studies Based on Approach to the Race or Ethnicity Variable

	Analytically informative for advancing racial health equity research		
	Yes	No	Total
Conceptually thoughtful about root causes of racial health inequities	Informative and thoughtful (n = 3 studies)^a^	Not informative, but thoughtful (n = 1)^b^	Thoughtful (n = 4)^c^
Not conceptually thoughtful about root causes of racial health inequities	Informative, not thoughtful (n = 18 studies)^d^	Not informative, not thoughtful (n = 22)^e^	Not thoughtful (n = 40)
Total	Informative (n = 21)	Not informative (n = 23)	Total n = 44)

^a^ Krieger et al,^28^ Towfighi et al,^161^ Szilagyi, et al.^165^

^b^ Crisanti et al.^142^

^c^ Krieger et al,^28^ Crisanti et al,^142^ Towfighi et al,^161^ Szilagyi, et al.^165^

^d^ Juillard et al,^26^ Mendelsohn et al,^29^ Tessaro et al,^47^ Slesnick et al,^62^ Kelley et al,^68^ Ziang et al,^78^ Glendenning-Napoli et al,^80^ Krieger et al,^87^ Chaiyachati et al,^93^ Krieger et al,^95^ Krieger et al,^96^ Lapham et al,^101^ Chan et al,^126^ Hilgeman et al,^128^ Foster et al,^151^ Lyles et al,^159^ Duncan et al,^170^ Whorms et al.^171^

^e^ Berkowitz et al,^23^ Morales et al,^30^ Seligman et al,^34^ Tomita et al,^35^ Liss et al,^58^ Gusmano et al,^63^ Duru et al,^66^ Lindau et al,^74^ Horwitz et al,^82^ Shah et al,^83^ Ciaranello et al,^85^ Chaiyachati et al,^92^ Melnikow et al,^94^ Nyamathi et al,^102^ Martinez et al,^117^ Guevara et al,^129^ Berkowitz et al,^135^ Tsai et al,^143^ Birkhead et al,^163^ Gottlieb et al,^167^ Moreno et al,^168^ Izumi et al.^169^

## Discussion

In this review based on PCORI’s scoping review and evidence map of social needs intervention studies, we developed and applied a simple framework of conceptual thoughtfulness and analytical informativeness to understand how social needs interventions may advance racial health equity. Our study yielded 2 key findings. First, fewer than one-third of the 152 studies in multiracial or multiethnic populations included race or ethnicity variables in their analyses of intervention effects (44 [28%]). Second, few studies (21 [14%]) conducted race or ethnicity–stratified analyses that were considered analytically informative for advancing health equity research. Even fewer (4 [9%]) provided conceptually thoughtful explanations for race as a proxy for root causes of racial health inequities and the reasons why we see differential outcomes by race or ethnicity.

Nearly 9 in 10 (86%) of the 152 studies in multiracial or multiethnic populations did not examine whether intervention effects differed by race or ethnicity. Because of the persistent and pervasive nature of racism, it is likely that social needs interventions operate differently in minoritized racial and ethnic populations. Failure to assess for differential outcomes by race or ethnicity prevents us from understanding whether minoritized racial and ethnic populations benefit from interventions at least as much as White populations prevent us from advancing our understanding of how social needs interventions can reduce racial or ethnic health inequities.^173,174^

Researchers may have failed to describe the rationale for using race or ethnicity in analyses for several possible reasons, including (1) limited awareness of the importance of doing so; (2) limited knowledge that racism, not race, is associated with social risks and poor health; and (3) scientific publishing norms that limit word counts and do not include standards for reporting on race or ethnicity. Corbie-Smith and colleagues’ qualitative research^175^ found that investigators did think critically about the use and implications of race in their research but did not consistently include this reflection in their published work. The same could have happened with the studies in this review. This suggests the need for continued education on the need to provide theory-driven conceptualizations of race and ethnicity and the risks of not doing so, as well as standard guidance on where such descriptions should be provided.

Our simple yet innovative 2-concept framework for assessing a study’s contributions to racial health equity research has several advantages. It is applicable to and can improve the design, conduct, and reporting of other areas of health services research where socially constructed variables are used in ways that imply that they are biological (eg, gender).

Our categorization framework can help individuals and groups that conduct systematic reviews by focusing on information with the highest utility for advancing racial health equity. For example, in 2021, the US Preventive Services Task Force (USPSTF) published 2 articles addressing racism in preventive services, with expectations for future USPSTF guideline recommendations.^176,177^ For systematic reviews that support clinical practice guideline development, routine synthesis of differences in effectiveness by race or ethnicity that do not consider analytical informativeness and conceptual thoughtfulness may exacerbate health inequities by perpetuating what has been termed *scientific racism*, or the belief that racial hierarchies are explained by biological differences.^178^ Our framework can be a useful addition to the next iteration of standards for reporting of systematic reviews on health equity (PRISMA extension on health equity).^11,12^

Our framework is consistent with and supports calls from multiple journals that have highlighted the problematic nature of imprecise definitions of race or ethnicity and failure to acknowledge structural racism as a fundamental cause of racial health inequities and have revised their author instructions accordingly.^179,180,181^ Changing the expectations of peer reviewers and journal editors about how race and racism are handled from conceptualization through data analyses and interpretation, and implications of the work, would facilitate this process.

### Limitations

A key limitation of our review is our inability to ascertain the myriad reasons why studies may not have conducted race- or ethnicity-stratified analysis (eg, sample size and power considerations) or may have chosen to conduct single race or ethnicity studies (eg, prior analyses and literature may have already demonstrated that a single racial or ethnic group has the greatest need and potential benefit from an intervention). Multiple factors likely influence and constrain authors’ ability to include more theory-informed conceptualizations of race and ethnicity in publications.

As part of our reliance on rapid review methods for searching and recheck of data for subgroup analyses, we may have missed potentially eligible studies. We conducted a single (rather than dual) risk-of-bias assessment. However, our analyses are not limited or constrained by the risk of bias of included studies, thereby limiting the impact of inaccuracies or inconsistencies in risk-of-bias ratings. Other decisions to streamline the review (focused data extraction, no strength-of-evidence grading, and a narrative synthesis) are not likely to have materially changed our findings because the review findings did not lend themselves to quantitative synthesis or stength-of-evidence grading.

## Conclusions

Structural racism is a fundamental cause of racial health inequities that disproportionately affect minoritized racial and ethnic groups and result in greater unmet social needs and risks than in White individuals. Consequently, social needs interventions should seek to reduce health inequities by race or ethnicity. Critical first steps in accomplishing this are understanding and explicitly acknowledging what race and ethnicity are serving as a proxy for. Our review of a scoping review found that studies of these interventions to date rarely offered conceptually thoughtful insight on the root causes for racial health inequities and infrequently conducted informative analyses on intervention effectiveness by race or ethnicity. Our findings pointed to a wide gap between expectations of these interventions’ potential to advance health equity and their design, conduct, and reporting. To advance the field, future work should use a theoretically sound conceptualization of how racism affects social drivers of health and use this understanding to inform methodological approaches to developing, implementing, and evaluating social needs interventions.

## Notes

Supplement 1. **eMethods.** Literature Search Strategies and Inclusion Criteria **eTable 1.** Ovid MEDLINE Search String and Yield for Food Insecurity, Housing, Education and Literacy, Financial Strain, Employment, Transportation, Utilities, Social Isolation, Early Childhood Development, Legal Services, and Childcare (November 29, 2021) **eTable 2.** Cochrane Library (Including Both CDSR and TRIALS) Search String and Yield for Food Insecurity, Housing, Education and Literacy, Financial Strain, Employment, Transportation, Utilities, Social Isolation, Early Childhood Development, Legal Services, and Childcare (November 29, 2021) **eTable 3.** Ovid MEDLINE Search String and Yield for Interpersonal Violence MEDLINE Search (November 29, 2021) **eTable 4.** Cochrane Library (Including Both CDSR and TRIALS) Search String and Yield for Interpersonal Violence (November 29, 2021) **eTable 5.** Ovid MEDLINE Search String and Yield for Access to Care MEDLINE Search (November 29, 2021) **eTable 6.** Cochrane Library (Including Both CDSR and CENTRAL) Search String and Yield for Access to Care (November 29, 2021) **eTable 7.** Systematic Reviews for Hand Searches (Last Search: November 29, 2021) **eTable 8.** Inclusion and Exclusion Criteria for Scoping and Rapid Reviews **eFigure 1.** Screening Approach for PCORI’s Scoping Review and Evidence Map **eAppendix 1.** Disposition of Studies Identified **eFigure 2.** Articles Included and Excluded for the Social Needs and Racial Health Equity Rapid Review **eAppendix 2.** Risk-of-Bias Assessment **eTable 9.** Individual Study Quality Assessment of Randomized Controlled Trials Based on Cochrane RoB 2.0 **eTable 10.** Individual Study Quality Assessment of Nonrandomized Studies of Interventions Using ROBINS-I **eTable 11.** Key Characteristics of Studies That Included Race or Ethnicity in Their Analyses **eTable 12.** Detailed Characteristics of Studies That Are Analytically Informative for Advancing Racial Health Equity Research (N = 21) **eTable 13.** Detailed Characteristics of Studies with Analyses That Are Not Informative for Advancing Racial Health Equity Research (N = 23)
eReferences

Supplement 2.
Data Sharing Statement
