# Supplementary material for: Racial Health Equity and Social Needs Interventions: A Review of a Scoping Review
Source: JAMA Netw Open. 2023 Jan 19;6(1):e2250654. doi: 10.1001/jamanetworkopen.2022.50654 (PMC9857687; doi:10.1001/jamanetworkopen.2022.50654)
Supplement: Supplement 1. — eMethods. Literature Search Strategies and Inclusion Criteria eTable 1. Ovid MEDLINE Search String and Yield for Food Insecurity, Housing, Education and Literacy, Financial Strain, Employment, Transportation, Utilities, Social Isolation, Early Childhood Development, Legal Services, and Childcare (November 29, 2021) eTable 2. Cochrane Library (Including Both CDSR and TRIALS) Search String and Yield for Food Insecurity, Housing, Education and Literacy, Financial Strain, Employment, Transportation, Utilities, Social Isolation, Early Childhood Development, Legal Services, and Childcare (November 29, 2021) eTable 3. Ovid MEDLINE Search String and Yield for Interpersonal Violence MEDLINE Search (November 29, 2021) eTable 4. Cochrane Library (Including Both CDSR and TRIALS) Search String and Yield for Interpersonal Violence (November 29, 2021) eTable 5. Ovid MEDLINE Search String and Yield for Access to Care MEDLINE Search (November 29, 2021) eTable 6. Cochrane Library (Including Both CDSR and CENTRAL) Search String and Yield for Access to Care (November 29, 2021) eTable 7. Systematic Reviews for Hand Searches (Last Search: November 29, 2021) eTable 8. Inclusion and Exclusion Criteria for Scoping and Rapid Reviews eFigure 1. Screening Approach for PCORI’s Scoping Review and Evidence Map eAppendix 1. Disposition of Studies Identified eFigure 2. Articles Included and Excluded for the Social Needs and Racial Health Equity Rapid Review eAppendix 2. Risk-of-Bias Assessment eTable 9. Individual Study Quality Assessment of Randomized Controlled Trials Based on Cochrane RoB 2.0 eTable 10. Individual Study Quality Assessment of Nonrandomized Studies of Interventions Using ROBINS-I eTable 11. Key Characteristics of Studies That Included Race or Ethnicity in Their Analyses eTable 12. Detailed Characteristics of Studies That Are Analytically Informative for Advancing Racial Health Equity Research (N = 21) eTable 13. Detailed Characteristics of Studies with Analyses That Are Not Inform [file jamanetwopen-e2250654-s001.pdf]

Supplemental Online Content

Cené CW, Viswanathan M, Fichtenberg CM, et al. Racial health equity and social needs interventions: a review of a scoping review. *JAMA Netw Open*. 2023;6(1):e2250654. doi:10.1001/jamanetworkopen.2022.50654

eMethods. Literature Search Strategies and Inclusion Criteria
eTable 1. Ovid MEDLINE Search String and Yield for Food Insecurity, Housing, Education and Literacy, Financial Strain, Employment, Transportation, Utilities, Social Isolation, Early Childhood Development, Legal Services, and Childcare (November 29, 2021)
eTable 2. Cochrane Library (Including Both CDSR and TRIALS) Search String and Yield for Food Insecurity, Housing, Education and Literacy, Financial Strain, Employment, Transportation, Utilities, Social Isolation, Early Childhood Development, Legal Services, and Childcare (November 29, 2021)
eTable 3. Ovid MEDLINE Search String and Yield for Interpersonal Violence MEDLINE Search (November 29, 2021)
eTable 4. Cochrane Library (Including Both CDSR and TRIALS) Search String and Yield for Interpersonal Violence (November 29, 2021)
eTable 5. Ovid MEDLINE Search String and Yield for Access to Care MEDLINE Search (November 29, 2021)
eTable 6. Cochrane Library (Including Both CDSR and CENTRAL) Search String and Yield for Access to Care (November 29, 2021)
eTable 7. Systematic Reviews for Hand Searches (Last Search: November 29, 2021)
eTable 8. Inclusion and Exclusion Criteria for Scoping and Rapid Reviews
eFigure 1. Screening Approach for PCORI's Scoping Review and Evidence Map
eAppendix 1. Disposition of Studies Identified
eFigure 2. Articles Included and Excluded for the Social Needs and Racial Health Equity Rapid Review
eAppendix 2. Risk-of-Bias Assessment
eTable 9. Individual Study Quality Assessment of Randomized Controlled Trials Based on Cochrane RoB 2.0
eTable 10. Individual Study Quality Assessment of Nonrandomized Studies of Interventions Using ROBINS-I
eTable 11. Key Characteristics of Studies That Included Race or Ethnicity in Their Analyses
eTable 12. Detailed Characteristics of Studies That Are Analytically Informative for Advancing Racial Health Equity Research (N=21)
eTable 13. Detailed Characteristics of Studies with Analyses That Are Not Informative for Advancing Racial Health Equity Research (N = 23)
eReferences

This supplemental material has been provided by the authors to give readers additional information about their work.

© 2023 Cené CW et al. *JAMA Network Open.*

eMethods. Literature Search Strategies and Inclusion Criteria

This supplement includes tables from the November 2021 data update for the PCORI scoping review and evidence map. To access prior search information, see the August 2021 scoping review and evidence map report at https://www.pcori.org/impact/evidence-maps-and-visualizations/social-needs-interventions-improve-health-outcomes.

eTable 1. Ovid MEDLINE Search String and Yield for Food Insecurity, Housing, Education and Literacy, Financial Strain, Employment, Transportation, Utilities, Social Isolation, Early Childhood Development, Legal Services, and Childcare (November 29, 2021)

Search	Query	Items Found
1	"Social Determinants of Health"/	4945
2	Social Conditions/	9489
3	Social Environment/	43 890
4	Social Class/	42 869
5	Socioeconomic Factors/	166 994
6	(social* adj1 determin*).ti,ab,kf.	9586
7	((determinant* or determinate*) adj2 health).ti,ab,kf.	11 010
8	((social* or socio*) adj1 condition*).ti,ab,kf.	7195
9	((social* or socio*) adj1 environment*).ti,ab,kf.	11 819
10	((social* or socio*) adj1 (factor* or gradient*)).ti,ab,kf.	40 858
11	((social* or socio*) adj1 (need* or require*)).ti,ab,kf.	2583
12	((social* or socio*) adj1 (equit* or inequit* or disparit* or equal* or inequal*)).ti,ab,kf.	9427
13	((social* or socio*) adj1 (hardship* or depriv* or challeng* or difficult* or barrier* or vulnerab* or disadvantag*)).ti,ab,kf.	13 894
14	((social* or socio*) adj1 risk*).ti,ab,kf.	2937
15	((social* or socio*) adj1 (status* or circumstance* or position* or class*)).ti,ab,kf.	64 922
16	Food Supply/	14 326
17	Hunger/	5738
18	(food adj2 (secur* or insecur* or unstable or stable or stabilit* or instabilit* or uncertain* or vulnerab* or hardship* or insufficien* or stress*)).ti,ab,kf.	11 420
19	food desert*.ti,ab,kf.	234
20	Housing/	18 941
21	Almshouses/	53
22	Public Housing/	1532
23	((hous* or home) adj3 (secur* or insecur* or unstable or stable or stabilit* or instabilit* or uncertain* or vulnerab* or hardship* or insufficien* or stress*)).ti,ab,kf.	5912
24	Homeless Persons/	8801
25	Homeless Youth/	1369
26	(homeless* or houseless*).ti,ab,kf.	10 711
27	Transportation/	11 058
28	Transportation Facilities/	59
29	Parking Facilities/	361
30	transportation*.ti.	3857
31	commut*.ti,ab,kf.	3647
32	Educational Status/	54 767
33	Academic Failure/	53
34	Literacy/	1120
35	Reading/	24 306
36	(literacy or literate or illitera*).ti,ab,kf.	24 732
37	(read* adj2 (proficien* or skill* or comprehension or level*)).ti,ab,kf.	7491
38	((education* or academic* or schola* or school*) adj2 (achieve* or status or attain* or equit* or inequit* or disparit* or equal* or inequalit* or level* or background*)).ti,ab,kf.	86 356

© 2023 Cené CW et al. *JAMA Network Open.*

Search	Query	Items Found
39	((education* or academic* or schola* or school*) adj2 (opportunit* or disadvantage* or advantage* or marginal* or disenfranchis* or vulnerab*)).ti,ab,kf.	4342
40	Poverty/	41 209
41	Poverty Areas/	6426
42	((economic* or income* or financ*) adj2 (achieve* or status or attain* or equit* or inequit* or disparit* or equal* or inequalit* or level* or background*)).ti,ab,kf.	34 522
43	((economic* or income* or financ*) adj2 (opportunit* or disadvantage* or advantage* or marginal* or disenfranchis* or vulnerab* or low or strain* or strugg* or stable or unstable or stabilit* or instabilit* or difficult* or problem*)).ti,ab,kf.	55 348
44	(poverty or indigent* or indigency or impoverish*).ti.	5579
45	Employment/	48 301
46	Unemployment/	7430
47	unemployment.ti,ab,kf.	10 690
48	unemployed.ti,ab,kf.	8488
49	underemploy*.ti,ab,kf.	355
50	(occupation* adj2 (status or level or class)).ti,ab,kf.	6659
51	jobless*.ti,ab,kf.	266
52	workless*.ti,ab,kf.	30
53	(employment adj2 (status or securit* or insecurit* or marginal* or precarious* or terminat*)).ti,ab,kf.	9722
54	Child Care/	5848
55	(child adj2 care).ti,ab,kf.	9965
56	Social Isolation/	15 223
57	(social* adj2 isolat*).ti,ab,kf.	9050
58	Legal Services/	41
59	(legal adj2 service*).ti,ab,kf.	682
60	((water or power or electric* or gas or sewer or sanit* or phone or internet or cable or satellite) adj3 (utility or utilities)).ti,ab,kf.	1303
61	Early Intervention, Educational/	3315
62	Child Development/	48 979
63	Language Development/	11 407
64	((child* or toddler or infant*) adj3 (educat* or develop*)).ti,ab,kf.	82 735
65	1 or 2 or 3 or 4 or 5 or 6 or 7 or 8 or 9 or 10 or 11 or 12 or 13 or 14 or 15 or 16 or 17 or 18 or 19 or 20 or 21 or 22 or 23 or 24 or 25 or 26 or 27 or 28 or 29 or 30 or 31 or 32 or 33 or 34 or 35 or 36 or 37 or 38 or 39 or 40 or 41 or 42 or 43 or 44 or 45 or 46 or 47 or 48 or 49 or 50 or 51 or 52 or 53	632 942
66	54 or 55 or 56 or 57 or 58 or 59 or 60 or 61 or 62 or 63 or 64	161 966
67	Mass Screening/	110 894
68	(Surveys and Questionnaires)	524 299
69	screen*.ti,ab,kf.	727 296
70	(instrument* or tool*).ti.	118 705
71	67 or 68 or 69 or 70	1 344 760
72	Needs Assessment/	31 907
73	Program Development/	30 084
74	(Referral and Consultation)	73 504
75	Pilot Projects/	135 699
76	Social Welfare/	9532
77	Food Assistance/	1475
78	Public Assistance/	2986
79	Patient Navigation/	900
80	Patient Advocacy/	24 073
81	Inservice Training/	20 668
82	Staff Development/	9764
83	intervention*.ti,ab,kf.	972 520
84	(need* adj2 (assessment* or evaluat* or determin*)).ti,ab,kf.	61 399
85	(food adj2 (assist* or aid or help*)).ti,ab,kf.	1374
86	((hous* or home) adj2 (assist* or aid or help*)).ti,ab,kf.	2558
87	(transportation adj2 (assist* or aid or help*)).ti,ab,kf.	231
88	((education* or academic* or schola* or school*) adj2 (assist* or aid or help*)).ti,ab,kf.	4339
89	((employment or occupation* or job*) adj2 (assist* or aid or help*)).ti,ab,kf.	1040

© 2023 Cené CW et al. *JAMA Network Open.*

Search	Query	Items Found
90	((economic* or income* or financ*) adj2 (assist* or aid or help*)).ti,ab,kf.	2589
91	patient navigat*.ti,ab,kf.	1037
92	patient advoca*.ti,ab,kf.	2455
93	((staff or employee*) adj2 (develop* or train* or educat* or curricul*)).ti,ab,kf.	15 273
94	((social* or socio* or communit* or neighbor* or neighbour*) adj3 (refer* or partner*)).ti,ab,kf.	12 372
95	((utility or utilities) adj2 (assist* or help or aid)).ti,ab,kf.	127
96	(legal adj2 (assist* or help or aid)).ti,ab,kf.	402
97	72 or 73 or 74 or 75 or 76 or 77 or 78 or 79 or 80 or 81 or 82 or 83 or 84 or 85 or 86 or 87 or 88 or 89 or 90 or 91 or 92 or 93 or 94	1 330 317
98	71 or 97	2 499 527
99	95 or 96	528
100	Primary Health Care/	85 557
101	Comprehensive Health Care/	6724
102	General Practice/	14 453
103	General Practitioners/	9206
104	Family Practice/	66 174
105	Physicians, Family/	16 806
106	Physicians, Primary Care/	4047
107	Primary Care Nursing/	544
108	Nurse Practitioners/	18 298
109	Family Nurse Practitioners/	64
110	Pediatric Nurse Practitioners/	176
111	Physician Assistants/	6006
112	Family Nursing/	1546
113	Community Health Nursing/	19 724
114	Community Health Centers/	7369
115	Community Mental Health Centers/	3015
116	Community Health Services/	32 618
117	Community Mental Health Services/	18 897
118	Community Health Workers/	5996
119	Safety-net Providers/	1155
120	primary care.ti,ab,kf.	113 504
121	primary health care.ti,ab,kf.	26 547
122	((family or general or primary) adj1 (medicine or practice or practitioner* or physician* or doctor* or provider* or clinic* or clinician*)).ti,ab,kf.	122 943
123	Patient-Centered Care/	21 528
124	Patient Care Team/	68 160
125	Health Services/	26 193
126	“Delivery of Health Care”/	102 744
127	Emergency Medicine/	14 443
128	Pediatric Emergency Medicine/	420
129	exp emergency medical services/	156 117
130	(emergency adj2 (medicine or servic* or room* or department* or physician* or doctor* or provider* or clinician*)).ti,ab,kf.	134 651
131	100 or 101 or 102 or 103 or 104 or 105 or 106 or 107 or 108 or 109 or 110 or 111 or 112 or 113 or 114 or 115 or 116 or 117 or 118 or 119 or 120 or 121 or 122 or 123 or 124 or 125 or 126 or 127 or 128 or 129 or 130	760 741
132	65 and 98 and 131	24 383
133	132 and “Case Reports”.sa_pubt.	166
134	132 not 133	24 217
135	limit 134 to english language	22 809
136	limit 135 to yr=“2020 -Current”	2558
137	98 or 99	2 499 873
138	66 and 131 and 137	3739
139	limit 138 to case reports	100
140	138 not 139	3639
141	limit 140 to english language	3419
142	limit 141 to yr=“2020 -Current”	353
143	136 or 142	2803

© 2023 Cené CW et al. *JAMA Network Open.*

Search	Query	Items Found
144	limit 143 to last year	2803

eTable 2. Cochrane Library (Including Both CDSR and TRIALS) Search String and Yield for Food Insecurity, Housing, Education and Literacy, Financial Strain, Employment, Transportation, Utilities, Social Isolation, Early Childhood Development, Legal Services, and Childcare (November 29, 2021)

Search	Query	Items Found
1	social*:ti,ab,kw near/1 determin*:ti,ab,kw	397
2	(determinant* or determinate*):ti,ab,kw near/2 health:ti,ab,kw	433
3	(social* or socio*):ti,ab,kw near/1 condition*:ti,ab,kw	396
4	(social* or socio*):ti,ab,kw near/1 environment*:ti,ab,kw	1668
5	(social* or socio*):ti,ab,kw near/1 (factor* or gradient*):ti,ab,kw	5318
6	(social* or socio*):ti,ab,kw near/1 (need* or require*):ti,ab,kw	248
7	(social* or socio*):ti,ab,kw near/1 (equit* or inequit* or disparit* or equal* or inequal*):ti,ab,kw	218
8	(social* or socio*):ti,ab,kw near/1 (hardship* or depriv* or challeng* or difficult* or barrier* or vulnerab* or disadvantag*):ti,ab,kw	1261
9	(social* or socio*):ti,ab,kw near/1 risk*:ti,ab,kw	264
10	(social* or socio*):ti,ab,kw near/1 (status* or circumstance* or position* or class* or standing):ti,ab,kw	5478
11	food*:ti,ab,kw near/2 (supply or secur* or insecur* or unstable or stable or stabilit* or instabilit* or uncertain* or vulnerab* or hardship* or insufficien* or stress*):ti,ab,kw	1039
12	food:ti,ab,kw next desert*:ti,ab,kw	10
13	(hous* or home):ti,ab,kw near/3 (secur* or insecur* or unstable or stable or stabilit* or instabilit* or uncertain* or vulnerab* or hardship* or insufficien* or stress*):ti,ab,kw	766
14	(homeless* or houseless*):ti,ab,kw	1053
15	Transportation*:ti,ab,kw	1780
16	commut*:ti,ab,kw	242
17	(literacy or literate or illitera*):ti,ab,kw	5566
18	read*:ti,ab,kw near/2 (proficien* or skill* or comprehension or level*):ti,ab,kw	969
19	(education* or academic* or schola* or school*):ti,ab,kw near/2 (achieve* or fail* or status or attain* or equit* or inequit* or disparit* or equal* or inequalit* or level* or background*):ti,ab,kw	10 522
20	(education* or academic* or schola* or school*):ti,ab,kw near/2 (opportunit* or disadvantage* or advantage* or marginal* or disenfranchis* or vulnerab*):ti,ab,kw	251
21	(economic* or income* or financ*):ti,ab,kw near/2 (achieve* or status or attain* or equit* or inequit* or disparit* or equal* or inequalit* or level* or background*):ti,ab,kw	2202
22	(economic* or income* or financ*):ti,ab,kw near/2 (opportunit* or disadvantage* or advantage* or marginal* or disenfranchis* or vulnerab* or low or strain* or strugg* or stable or unstable or stabilit* or instabilit* or difficult* or problem* or stress*):ti,ab,kw	6660
23	(poverty or indigent* or indigency or impoverish*):ti,ab,kw	3274
24	unemployment:ti,ab,kw	822
25	unemployed:ti,ab,kw	637
26	underemployed:ti,ab,kw	10
27	(occupation* or job):ti,ab,kw near/2 (status or level or class):ti,ab,kw	444
28	jobless*:ti,ab,kw	6
29	workless*:ti,ab,kw	2
30	(employment or job or occupation*):ti,ab,kw near/2 (status or securit* or insecurit* or marginal* or precarious* or terminat*):ti,ab,kw	1345
31	child:ti,ab,kw near/2 care:ti,ab,kw	3990
32	social*:ti,ab,kw near/2 isolat*:ti,ab,kw	1074
33	legal:ti,ab,kw near/2 service*:ti,ab,kw	30
34	(water or power or electric* or gas or sewer or sanit* or phone or internet or cable or satellite):ti,ab,kw near/3 (utility or utilities):ti,ab,kw	66
35	(child* or toddler or infant*):ti,ab,kw near/3 (educat* or develop* or language*):ti,ab,kw	12 934

© 2023 Cené CW et al. *JAMA Network Open.*

Search	Query	Items Found
36	#5 OR #6 OR #7 OR #8 OR #9 OR #10 OR #11 OR #12 OR #13 OR #14 OR #15 OR #16 OR #17 OR #18 OR #19 OR #20 OR #21 OR #22 OR #23 OR #24 OR #25 OR #26 OR #27 OR #28 OR #29 OR #30	38 623
37	#31 OR #32 OR #33 OR #34 OR #35	17 389
38	screen*:ti,ab,kw	81 319
39	(instrument* or tool*):ti	6419
40	#38 OR #39	87 060
41	intervention*:ti,ab,kw	453 555
42	need*:ti,ab,kw near/2 (assessment* or evaluat* or determin*):ti,ab,kw	9355
43	program*:ti,ab,kw near/2 develop*:ti,ab,kw	4252
44	pilot:ti,ab,kw next project*:ti,ab,kw	22 015
45	food:ti,ab,kw near/2 (assist* or aid or help*):ti,ab,kw	294
46	(hous* or home):ti,ab,kw near/2 (assist* or aid or help*):ti,ab,kw	425
47	transportation*:ti,ab,kw near/2 (assist* or aid or help*):ti,ab,kw	35
48	(education* or academic* or schola* or school*):ti,ab,kw near/2 (assist* or aid or help*):ti,ab,kw	820
49	(employment or occupation* or job*):ti,ab,kw near/2 (assist* or aid or help*):ti,ab,kw	99
50	(economic* or income* or financ*):ti,ab,kw near/2 (assist* or aid or help*):ti,ab,kw	317
51	patient*:ti,ab,kw near/1 navigat*:ti,ab,kw	640
52	patient*:ti,ab,kw near/2 advoca*:ti,ab,kw	378
53	(staff or employee*):ti,ab,kw near/2 (develop* or train* or educat* or curricul*):ti,ab,kw	2788
54	(social* or socio* or communit* or neighbor* or neighbour*):ti,ab,kw near/3 (refer* or partner*):ti,ab,kw	1647
55	(utility or utilities):ti,ab,kw near/2 (assist* or help or aid):ti,ab,kw	12
56	legal:ti,ab,kw near/2 (assist* or help or aid):ti,ab,kw	21
57	#41 OR #42 OR #43 OR #44 OR #45 OR #46 OR #47 OR #48 OR #49 OR #50 OR #51 OR #52 OR #53 OR #54 OR #55 OR #56	476 950
58	#40 OR #57	523 172
59	#36 AND #58	24 902
60	#37 AND #58	10 076
61	primary:ti,ab,kw next care:ti,ab,kw	19 802
62	comprehensive:ti,ab,kw next care:ti,ab,kw	327
63	“primary health care”:ti,ab,kw	7097
64	“comprehensive health care”:ti,ab,kw	99
65	comprehensive:ti,ab,kw next healthcare:ti,ab,kw	16
66	primary:ti,ab,kw next healthcare:ti,ab,kw	773
67	(safety-net:ti,ab,kw or “safety net”:ti,ab,kw) next clinic*:ti,ab,kw	79
68	“community health center”:ti,ab,kw	302
69	“community health centers”:ti,ab,kw	669
70	“federally qualified health center”:ti,ab,kw	148
71	“federally qualified health centers”:ti,ab,kw	136
72	fqhc:ti,ab,kw	91
73	(family or general or primary):ti,ab,kw near/2 (medicine or practice or practitioner* or physician* or doctor* or provider* or clinic* or clinician* or nurs*):ti,ab,kw	29 576
74	emergency:ti,ab,kw near/2 (medicine or servic* or room* or department* or physician* or doctor* or provider* or clinician*):ti,ab,kw	18 114
75	#61 OR #62 OR #63 OR #64 OR #65 OR #66 OR #67 OR #68 OR #69 OR #70 OR #71 OR #72 OR #73 OR #74	58 691
76	#59 AND #75 with Cochrane Library publication date Between Jan 2019 and Dec 2021	1429
77	#60 AND #75 with Cochrane Library publication date Between Jan 2017 and Dec 2021	616
78	#76 OR #77 with Cochrane Library publication date in the last year	390

eTable 3. Ovid MEDLINE Search String and Yield for Interpersonal Violence MEDLINE Search (November 29, 2021)

Search	Query	Items Found
1	Physical Abuse/	922
2	Gun Violence/	341

© 2023 Cené CW et al. *JAMA Network Open.*

Search	Query	Items Found
3	gender based violence	1143
4	Elder Abuse	2909
5	rape	10 720
6	workplace violence	1870
7	torture	2763
8	((elder* or geriatric* or aged or interpersonal or gun* or workplace) adj2 (violen* or abus* or neglect* or maltreat* or batter*)).ti.	2899
9	1 or 2 or 3 or 4 or 5 or 6 or 7 or 8	21 066
10	Mass Screening	113 334
11	Anonymous Testing	543
12	Mass Chest X-Ray	1957
13	Multiphasic Screening	1159
14	risk	2 533 564
15	logistic models	150 585
16	Protective Factors	14 862
17	Risk Assessment	322 908
18	Adverse Outcome Pathways	502
19	“Healthcare Failure Mode and Effect Analysis”	180
20	Risk Factors	1 075 444
21	(screen* or risk).ti.	611 614
22	10 or 11 or 12 or 13 or 14 or 15 or 16 or 17 or 18 or 19 or 20 or 21	2 766 973
23	9 and 22	4919
24	exp “Surveys and Questionnaires”/	1 139 906
25	exp Diagnosis/	9 051 301
26	interven*.ti.	149 727
27	24 or 25 or 26	9 797 601
28	9 and 27	4882
29	23 or 28	8085
30	limit 29 to english language	7602
31	limit 30 to last year	996

eTable 4. Cochrane Library (Including Both CDSR and TRIALS) Search String and Yield for Interpersonal Violence (November 29, 2021)

Search	Query	Items Found
1	physical abuse	2415
2	gun violence	43
3	gender based violence	473
4	elder abuse	999
5	rape	430
6	workplace violence	78
7	torture	86
8	((elder* or geriatric* or aged or interpersonal or gun* or workplace) NEAR/2 (violen* or abus* or neglect* or maltreat* or batter*)):ti	106
9	#1 OR #2 OR #3 OR #4 OR #5 OR #6 OR #7 OR #8	3807
10	Mass Screening	9648
11	Anonymous Testing	334
12	Mass Chest X-Ray	502
13	Multiphasic Screening	58
14	risk	260 746
15	logistic models	10 074
16	Protective Factors	4764
17	Risk Assessment	75 526
18	Adverse Outcome Pathways	1668
19	“Healthcare Failure Mode and Effect Analysis”	2
20	Risk Factors	84 244
21	(screen* OR risk*):ti	55 968
22	10 OR 11 OR 12 OR 13 OR 14 OR 15 OR 16 OR 17 OR 18 OR 19 OR 20 OR 21	279 744

Search	Query	Items Found
23	9 AND 22	1853
24	MeSH descriptor: [Surveys and Questionnaires] explode all trees	57 574
25	MeSH descriptor: [Diagnosis] explode all trees	350 855
26	interven*:ti	67 128
27	24 OR 25 OR 26	425 559
28	9 AND 27	1098
29	23 OR 28 with Cochrane Library publication date in the last year	200

eTable 5. Ovid MEDLINE Search String and Yield for Access to Care MEDLINE Search (November 29, 2021)

Search	Query	Items Found
1	Social Determinants of Health	8691
2	Social Conditions	11 664
3	Social Environment	48 121
4	Social Class	47 476
5	Socioeconomic Factors	172 577
6	(social* adj1 determin*).ti,ab,kf.	9586
7	((determinant* or determinate*) adj2 health).ti,ab,kf.	11 010
8	((social* or socio*) adj1 condition*).ti,ab,kf.	7195
9	((social* or socio*) adj1 environment*).ti,ab,kf.	11 819
10	((social* or socio*) adj1 (factor* or gradient*)).ti,ab,kf.	40 858
11	((social* or socio*) adj1 (need* or require*)).ti,ab,kf.	2583
12	((social* or socio*) adj1 (equit* or inequit* or disparit* or equal* or inequal*)).ti,ab,kf.	9427
13	((social* or socio*) adj1 (hardship* or depriv* or challeng* or difficult* or barrier* or vulnerab* or disadvantag*)).ti,ab,kf.	13 894
14	((social* or socio*) adj1 risk*).ti,ab,kf.	2937
15	((social* or socio*) adj1 (status* or circumstance* or position* or class* or standing)).ti,ab,kf.	65 196
16	1 or 2 or 3 or 4 or 5 or 6 or 7 or 8 or 9 or 10 or 11 or 12 or 13 or 14 or 15	326 730
17	Early Intervention, Educational	3316
18	Internet-Based Intervention	1095
19	Early Medical Intervention	3545
20	Needs Assessment	35 341
21	Program Development	31 957
22	(Referral and Consultation)	73 504
23	Pilot Projects	136 253
24	Social Welfare	11 610
25	Patient Navigation	1231
26	Patient Advocacy	24 870
27	Inservice Training	20 788
28	Staff Development	10 812
29	intervention*.ti,ab,kf.	972 520
30	(need* adj2 (assessment* or evaluat* or determin*)).ti,ab,kf.	61 399
31	patient navigat*.ti,ab,kf.	1037
32	patient advoca*.ti,ab,kf.	2455
33	((staff or employee*) adj2 (develop* or train* or educat* or curricul*)).ti,ab,kf.	15 273
34	((social* or socio* or communit* or neighbor* or neighbour*) adj3 (refer* or partner*)).ti,ab,kf.	12 372
35	17 or 18 or 19 or 20 or 21 or 22 or 23 or 24 or 25 or 26 or 27 or 28 or 29 or 30 or 31 or 32 or 33 or 34	1 324 580
36	Health Services Accessibility	81 817
37	Health Equity	5150
38	Right to Health	1461
39	Universal Health Care	1202
40	(primary care adj3 (access* or avail* or utiliz*)).ti,ab,kf.	3259
41	(health services adj3 (access* or avail* or utiliz*)).ti,ab,kf.	8696
42	(healthcare adj3 (access* or avail* or utiliz*)).ti,ab,kf.	15 438

© 2023 Cené CW et al. *JAMA Network Open.*

Search	Query	Items Found
43	(health care adj3 (access* or avail* or utiliz*)).ti,ab,kf.	24 667
44	36 or 37 or 38 or 39 or 40 or 41 or 42 or 43	123 885
45	Primary Health Care	97 503
46	Comprehensive Health Care	7355
47	General Practice	47 517
48	General Practitioners	38 250
49	Family Practice/	66 174
50	Physicians, Family/	16 806
51	Physicians, Primary Care/	4047
52	Primary Care Nursing/	544
53	Nurse Practitioners/	18 298
54	Family Nurse Practitioners/	64
55	Pediatric Nurse Practitioners/	176
56	Physician Assistants/	6006
57	Family Nursing/	1546
58	Community Health Nursing/	19 724
59	Community Health Centers/	7369
60	Community Mental Health Centers/	3015
61	Community Health Services/	32 618
62	Community Mental Health Services/	18 897
63	Community Health Workers/	5996
64	Safety-net Providers/	1155
65	primary care.ti,ab,kf.	113 504
66	primary health care.ti,ab,kf.	26 547
67	((family or general or primary) adj1 (medicine or practice or practitioner* or physician* or doctor* or provider* or clinic* or clinician*)).ti,ab,kf.	122 943
68	36 or 37 or 38 or 39 or 40 or 41 or 42 or 43 or 44 or 45 or 46 or 47 or 48 or 49 or 50 or 51 or 52 or 53 or 54 or 55 or 56 or 57 or 58 or 59 or 60 or 61 or 62 or 63 or 64 or 65 or 66 or 67	487 113
69	16 and 35 and 68	9381
70	limit 69 to (yr="1995 -Current" and (systematic reviews pre 2019 or systematic reviews))	483
71	(systematic adj3 (review or assess* or eval*)).ti.	137 531
72	69 and 71	177
73	70 or 72	484
74	35 and 44	27 559
75	limit 74 to (yr="1995 -Current" and (systematic reviews pre 2019 or systematic reviews))	1904
76	71 and 74	732
77	75 or 76	1908
78	73 or 77	2103
79	Social Determinants of Health	8691
80	Social Conditions	11 664
81	Social Environment	48 121
82	Social Class	47 476
83	Socioeconomic Factors	172 577
84	(social* adj1 determin*).ti,ab,kf.	9586
85	((determinant* or determinate*) adj2 health).ti,ab,kf.	11 010
86	((social* or socio*) adj1 condition*).ti,ab,kf.	7195
87	((social* or socio*) adj1 environment*).ti,ab,kf.	11 819
88	((social* or socio*) adj1 (factor* or gradient*)).ti,ab,kf.	40 858
89	((social* or socio*) adj1 (need* or require*)).ti,ab,kf.	2583
90	((social* or socio*) adj1 (equit* or inequit* or disparit* or equal* or inequal*)).ti,ab,kf.	9427
91	((social* or socio*) adj1 (hardship* or depriv* or challeng* or difficult* or barrier* or vulnerab* or disadvantag*)).ti,ab,kf.	13 894
92	((social* or socio*) adj1 risk*).ti,ab,kf.	2937
93	((social* or socio*) adj1 (status* or circumstance* or position* or class* or standing)).ti,ab,kf.	65 196
94	79 or 80 or 81 or 82 or 83 or 84 or 85 or 86 or 87 or 88 or 89 or 90 or 91 or 92 or 93	326 730
95	Early Intervention, Educational	3316

© 2023 Cené CW et al. *JAMA Network Open.*

Search	Query	Items Found
96	Internet-Based Intervention	1095
97	Early Medical Intervention	3545
98	Needs Assessment	35 341
99	Program Development	31 957
100	(Referral and Consultation)	73 504
101	Pilot Projects	136 253
102	Social Welfare	11 610
103	Patient Navigation	1231
104	Patient Advocacy	24 870
105	Inservice Training	20 788
106	Staff Development	10 812
107	intervention*.ti,ab,kf.	972 520
108	(need* adj2 (assessment* or evaluat* or determin*)).ti,ab,kf.	61 399
109	patient navigat*.ti,ab,kf.	1037
110	patient advoca*.ti,ab,kf.	2455
111	((staff or employee*) adj2 (develop* or train* or educat* or curricul*)).ti,ab,kf.	15 273
112	((social* or socio* or communit* or neighbor* or neighbour*) adj3 (refer* or partner*)).ti,ab,kf.	12 372
113	95 or 96 or 97 or 98 or 99 or 100 or 101 or 102 or 103 or 104 or 105 or 106 or 107 or 108 or 109 or 110 or 111 or 112	1 324 580
114	Health Services Accessibility	81 817
115	Health Equity	5150
116	Right to Health	1461
117	Universal Health Care	1202
118	(primary care adj3 (access* or avail* or utiliz*)).ti,ab,kf.	3259
119	(health services adj3 (access* or avail* or utiliz*)).ti,ab,kf.	8696
120	(healthcare adj3 (access* or avail* or utiliz*)).ti,ab,kf.	15 438
121	(health care adj3 (access* or avail* or utiliz*)).ti,ab,kf.	24 667
122	114 or 115 or 116 or 117 or 118 or 119 or 120 or 121	123 885
123	Primary Health Care	97 503
124	Comprehensive Health Care	7355
125	General Practice	47 517
126	General Practitioners	38 250
127	Family Practice/	66 174
128	Physicians, Family/	16 806
129	Physicians, Primary Care/	4047
130	Primary Care Nursing/	544
131	Nurse Practitioners/	18 298
132	Family Nurse Practitioners/	64
133	Pediatric Nurse Practitioners/	176
134	Physician Assistants/	6006
135	Family Nursing/	1546
136	Community Health Nursing/	19 724
137	Community Health Centers/	7369
138	Community Mental Health Centers/	3015
139	Community Health Services/	32 618
140	Community Mental Health Services/	18 897
141	Community Health Workers/	5996
142	Safety-net Providers/	1155
143	primary care.ti,ab,kf.	113 504
144	primary health care.ti,ab,kf.	26 547
145	((family or general or primary) adj1 (medicine or practice or practitioner* or physician* or doctor* or provider* or clinic* or clinician*)).ti,ab,kf.	122 943
146	114 or 115 or 116 or 117 or 118 or 119 or 120 or 121 or 122 or 123 or 124 or 125 or 126 or 127 or 128 or 129 or 130 or 131 or 132 or 133 or 134 or 135 or 136 or 137 or 138 or 139 or 140 or 141 or 142 or 143 or 144 or 145	487 113
147	94 and 113 and 146	9381
148	limit 147 to (yr="1995 -Current" and (systematic reviews pre 2019 or systematic reviews))	483
149	(systematic adj3 (review or assess* or eval*)).ti.	137 531

© 2023 Cené CW et al. *JAMA Network Open.*

Search	Query	Items Found
150	147 and 149	177
151	148 or 150	484
152	113 and 122	27 559
153	limit 152 to (yr="1995 -Current" and (systematic reviews pre 2019 or systematic reviews))	1904
154	149 and 152	732
155	153 or 154	1908
156	151 or 155	2103
157	Limit to last year	129

e Table 6. Cochrane Library (Including Both CDSR and CENTRAL) Search String and Yield for Access to Care (November 29, 2021)

Search	Query	Items Found
1	social*:ti,ab,kw near/1 determin*:ti,ab,kw	397
2	(determinant* or determinate*):ti,ab,kw near/2 health:ti,ab,kw	433
3	(social* or socio*):ti,ab,kw near/1 condition*:ti,ab,kw	396
4	(social* or socio*):ti,ab,kw near/1 environment*:ti,ab,kw	1668
5	(social* or socio*):ti,ab,kw near/1 (factor* or gradient*):ti,ab,kw	5318
6	(social* or socio*):ti,ab,kw near/1 (need* or require*):ti,ab,kw	248
7	(social* or socio*):ti,ab,kw near/1 (equit* or inequit* or disparit* or equal* or inequal*):ti,ab,kw	218
8	(social* or socio*):ti,ab,kw near/1 (hardship* or depriv* or challeng* or difficult* or barrier* or vulnerab* or disadvantag*):ti,ab,kw	1261
9	(social* or socio*):ti,ab,kw near/1 risk*:ti,ab,kw	264
10	(social* or socio*):ti,ab,kw near/1 (status* or circumstance* or position* or class* or standing):ti,ab,kw	5478
11	#1 OR #2 OR #3 OR #4 OR #5 OR #6 OR #7 OR #8 OR #9 OR #10	13 862
12	intervention*:ti,ab,kw	453 553
13	need*:ti,ab,kw near/2 (assessment* or evaluat* or determin*):ti,ab,kw	9355
14	program*:ti,ab,kw near/2 develop*:ti,ab,kw	4252
15	pilot:ti,ab,kw next project*:ti,ab,kw	22 015
16	patient*:ti,ab,kw near/1 navigat*:ti,ab,kw	640
17	patient*:ti,ab,kw near/2 advoca*:ti,ab,kw	378
18	(staff or employee*):ti,ab,kw near/2 (develop* or train* or educat* or curricul*):ti,ab,kw	2788
19	(social* or socio* or communit* or neighbor* or neighbour*):ti,ab,kw near/3 (refer* or partner*):ti,ab,kw	1647
20	#12 OR #13 OR #14 OR #15 OR #16 OR #17 OR #18 OR #19	476 271
21	"primary care":ti,ab,kw near/3 (access* or avail* or utiliz*or utilis*):ti,ab,kw	318
22	"health services":ti,ab,kw near/3 (access* or avail* or utiliz*or utilis*):ti,ab,kw	1188
23	healthcare:ti,ab,kw near/3 (access* or avail* or utiliz*or utilis*):ti,ab,kw	962
24	"health care":ti,ab,kw near/3 (access* or avail* or utiliz*or utilis*):ti,ab,kw	1543
25	#21 OR #22 OR #23 OR #24	3696
26	primary:ti,ab,kw next care:ti,ab,kw	19 802
27	comprehensive:ti,ab,kw next care:ti,ab,kw	327
28	"primary health care":ti,ab,kw	7097
29	"comprehensive health care":ti,ab,kw	99
30	comprehensive:ti,ab,kw next healthcare:ti,ab,kw	16
31	primary:ti,ab,kw next healthcare:ti,ab,kw	773
32	(safety-net:ti,ab,kw or "safety net":ti,ab,kw) next clinic*:ti,ab,kw	79
33	"community health center":ti,ab,kw	302
34	"community health centers":ti,ab,kw	669
35	"federally qualified health center":ti,ab,kw	148
36	"federally qualified health centers":ti,ab,kw	136
37	fqhc:ti,ab,kw	91
38	(family or general or primary):ti,ab,kw near/2 (medicine or practice or practitioner* or physician* or doctor* or provider* or clinic* or clinician* or nurs*):ti,ab,kw	29 576
39	26 OR 27 OR 28 OR 29 OR 30 OR 31 OR 32 OR 33 OR 34 OR 35 OR 36 OR 37 OR 38	42 224

© 2023 Cené CW et al. *JAMA Network Open.*

Search	Query	Items Found
40	25 OR 39	44 848
41	11 AND 20 AND 40	1200
42	20 AND 25	2473
43	41 OR 42 with Cochrane Library publication date in the last year	330

eTable 7. Systematic Reviews for Hand Searches (Last Search: November 29, 2021)

1.	Allen LN, Smith RW, Simmons-Jones F, et al. Addressing social determinants of noncommunicable diseases in primary care: a systematic review. *Bull World Health Organ*. 2020;98(11):754-765. doi:10.2471/BLT.19.248278. PMID: 33177772
2.	Aubry T, Goering P, Veldhuizen S, et al. A multiple-city RCT of housing first with assertive community treatment for homeless Canadians with serious mental illness. *Psychiatr Serv*. 2016;67(3):275-281. doi:10.1176/appi.ps.20140058710.1176/appi.ps.201400587. PMID: 26620289
3.	Avancena ALV, Prosser LA. Examining equity effects of health interventions in cost-effectiveness analysis: a systematic review. *Value Health*. 2021;24(1):136-143. doi:10.1016/j.jval.2020.10.010
4.	Baxter AJ, Tweed EJ, Katikireddi SV, et al. Effects of housing first approaches on health and well-being of adults who are homeless or at risk of homelessness: systematic review and meta-analysis of randomised controlled trials. *J Epidemiol Community Health*. 2019;73(5):379-387. doi:10.1136/jech-2018-210981. PMID: 30777888
5.	Boch S, Keedy H, Chavez L, et al. An integrative review of social determinants of health screenings used in primary care settings. *J Health Care Poor Underserved*. 2020;31(2):603-622. doi:10.1353/hpu.2020.0048. PMID: 33410796
6.	Bou Malham C, El Khatib S, Cestac P, Andrieu S, Rouch L, Salameh P. Impact of pharmacist-led interventions on patient care in ambulatory care settings: a systematic review. *Int J Clin Pract*. 2021;75(11):e14864. doi:10.1111/ijcp.14864
7.	Brush BL, Mentz G, Jensen M, et al. Success in long-standing community-based participatory research (CBPR) partnerships: a scoping literature review. *Health Educ Behav*. 2020;47(4):556-568. doi:10.1177/1090198119882989
8.	Budde H, Williams GA, Winkelmann J, Pfirter L, Maier CB. The role of patient navigators in ambulatory care: overview of systematic reviews. *BMC Health Serv Res*. 2021;21(1):1166. doi:10.1186/s12913-021-07140-6
9	Burns J, Conway DI, Gnich W, Macpherson LMD. A systematic review of interventions to link families with preschool children from healthcare services to community-based support. *J Public Health (Oxf)*. 2021;43(2):e224-e235. doi:10.1093/pubmed/fdaa242
10.	Byon HD, Lee M, Choi M, Sagherian K, Crandall M, Lipscomb J. Prevalence of type II workplace violence among home healthcare workers: a meta-analysis. *Am J Ind Med*. 2020;63(5):442-455. doi:10.1002/ajim.23095
11.	Choi KR, Easterlin MC. Intervention models for increasing access to behavioral health services among youth: a systematic review. *J Dev Behav Pediatr*. 2018;39(9):754-762. doi:10.1097/DBP.0000000000000623. PMID: 30334855
12.	Davidson KW, Krist AH, Tseng CW, et al. Incorporation of social risk in US Preventive Services Task Force recommendations and identification of key challenges for primary care. *JAMA*. 2021;326(14):1410-1415. doi:10.1001/jama.2021.12833
13.	Eder M, Henninger M, Durbin S, et al. Screening and interventions for social risk factors: technical brief to support the US Preventive Services Task Force. *JAMA*. 2021;326(14):1416-1428. doi:10.1001/jama.2021.12825
14.	Evans TS, Berkman N, Brown C, et al. *Disparities Within Serious Mental Illness*. Agency for Healthcare Research and Quality; 2016. Report No.: 16-EHC027-EF. 2016. PMID: 27336120
15.	Ezell JM. Understanding the situational context for interpersonal violence: a review of individual-level attitudes, attributions, and triggers. *Trauma Violence Abuse*. 2021;22(3):571-587. doi:10.1177/1524838019869100
16.	Fitzpatrick-Lewis D, Ganann R, Krishnaratne S, et al. Effectiveness of interventions to improve the health and housing status of homeless people: a rapid systematic review. *BMC Public Health*. 2011;11:638. doi:10.1186/1471-2458-11-638. PMID: 21831318
17.	Formosa EA, Kishimoto V, Orchanian-Cheff A, Hayman K. Emergency department interventions for homelessness: a systematic review. *CJEM*. 2021;23(1):111-122. doi:10.1007/s43678-020-00008-4
18.	Garg A, Brochier A, Messmer E, Fiori KP. Clinical approaches to reducing material hardship due to poverty: social risks/needs identification and interventions. *Acad Pediatr*. 2021;21(8S):S154-S160. doi:10.1016/j.acap.2021.02.007

© 2023 Cené CW et al. *JAMA Network Open.*

19.	Garvin LA, Pugatch M, Gurewich D, Pendergast JN, Miller CJ. Interorganizational care coordination of rural veterans by veterans affairs and community care programs: a systematic review. *Med Care*. 2021;59(suppl 3):S259-S269. doi:10.1097/MLR.0000000000001542
20.	Ghanbarzadegan A, Balasubramanian M, Luzzi L, Brennan D, Bastani P. Inequality in dental services: a scoping review on the role of access toward achieving universal health coverage in oral health. *BMC Oral Health*. 2021;21(1):404. doi:10.1186/s12903-021-01765-z
21.	Hand T, Rosseau NA, Stiles CE, et al. The global role, impact, and limitations of community health workers (CHWs) in breast cancer screening: a scoping review and recommendations to promote health equity for all. *Glob Health Action*. 2021;14(1):1883336. doi:10.1080/16549716.2021.1883336
22.	Hasan M, Singh H, Haffizulla F. Culturally sensitive health education in the Caribbean diaspora: a scoping review. *Int J Environ Res Public Health*. 2021;18(4):04. doi:10.3390/ijerph18041476
23.	Health Quality Ontario. Interventions to improve access to primary care for people who are homeless: a systematic review. *Ont Health Technol Assess Ser*. 2016;16(9):1-50. PMID: 27099645
24.	Hopman P, de Bruin SR, Forjaz MJ, et al. Effectiveness of comprehensive care programs for patients with multiple chronic conditions or frailty: a systematic literature review. *Health Policy*. 2016;120(7):818-832. doi:10.1016/j.healthpol.2016.04.002. PMID: 27114104
25.	Huhtakangas M, Tuomikoski AM, Kyngas H, Kanste O. Frequent attenders' experiences of encounters with healthcare personnel: a systematic review of qualitative studies. *Nurs Health Sci*. 2021;23(1):53-68. doi:10.1111/nhs.12784
26.	Jack HE, Arabadjis SD, Sun L, et al. Impact of community health workers on use of health care services in the United States: a systematic review. *J Gen Intern Med*. 2017;32(3):325-344. doi:10.1007/s11606-016-3922-9. PMID: 27921257
27.	Jones T, Luth EA, Lin SY, Brody AA. Advance care planning, palliative care, and end-of-life care interventions for racial and ethnic underrepresented groups: a systematic review. *Pain Symptom Manage*. 2021;62(3):e248-e260. doi:10.1016/j.jpainsymman.2021.04.025
28.	Kaur H, Saad A, Magwood O, et al. Understanding the health and housing experiences of refugees and other migrant populations experiencing homelessness or vulnerable housing: a systematic review using GRADE-CERQual. *CMAJ Open*. 2021;9(2):E681-E692. doi:10.9778/cmajo.20200109
29.	Kehle SM, Greer N, Rutks I, et al. Interventions to improve veterans' access to care: a systematic review of the literature. *J Gen Intern Med*. 2011;26(suppl 2):689-696. doi:10.1007/s11606-011-1849-8. PMID: 21989623
30.	Khanassov V, Pluye P, Descoteaux S, et al. Organizational interventions improving access to community-based primary health care for vulnerable populations: a scoping review. *Int J Equity Health*. 2016;15(1):168. PMID: 27724952
31.	League A, Donato KM, Sheth N, et al. A systematic review of medical-legal partnerships serving immigrant communities in the United States. *J Immigr Minor Health*. 2021;23(1):163-174. doi:10.1007/s10903-020-01088-1
32.	Luchenski S, Maguire N, Aldridge RW, et al. What works in inclusion health: overview of effective interventions for marginalised and excluded populations. *Lancet*. 2018;391(10117):266-280. doi:10.1016/S0140-6736(17)31959-1. PMID: 29137868
33.	Macedo CM, Egry EY. Conceptual frameworks for programs addressing violence against children: a scoping review. Review. *Rev Esc Enferm USP*. 2021;55:e20200182. doi:10.1590/1980-220X-REEUSP-2020-0182
34.	Machado AA, Edwards SA, Mueller M, Saini V. Effective interventions to increase routine childhood immunization coverage in low socioeconomic status communities in developed countries: a systematic review and critical appraisal of peer-reviewed literature. *Vaccine*. 2021;39(22):2938-2964. doi:10.1016/j.vaccine.2021.03.088
35.	Marcellus L, MacKinnon K, Gordon C, Shaw L. Interventions and programs that support the health and development of infants with prenatal substance exposure in foster care: a scoping review. *JBI Evid Synth*. 2021;19(8):1844-1886. doi:10.11124/JBIES-20-00071
36.	Martinez GS, Chu J, Marachelian A, et al. More than health care: the value of addressing health, education, and social service needs together through community health centers. *J Ambul Care Manage*. 2020;43(1):41-54. doi:10.1097/JAC.0000000000000314. PMID: 31770185
37.	Miler JA, Carver H, Foster R, et al. Provision of peer support at the intersection of homelessness and problem substance use services: a systematic "state of the art" review. *BMC Public Health*. 2020;20(1):641. doi:10.1186/s12889-020-8407-4. PMID: 32381086
38.	Miler JA, Carver H, Masterton W, et al. What treatment and services are effective for people who are homeless and use drugs? A systematic 'review of reviews.' *PLoS ONE*. 2021;16(7):e0254729. doi:10.1371/journal.pone.0254729
39.	Moen M, Storr C, German D, Friedmann E, Johantgen M. A review of tools to screen for social determinants of health in the United States: a practice brief. *Popul Health Manag*. 2020;23(6):422-429. doi:10.1089/pop.2019.0158

© 2023 Cené CW et al. *JAMA Network Open.*

40.	O'Brien J, Fossey E, Palmer VJ. A scoping review of the use of co-design methods with culturally and linguistically diverse communities to improve or adapt mental health services. *Health Soc Care Community*. 2021;29(1):1-17. doi:10.1111/hsc.13105
41.	Parry J, Vanstone M, Grignon M, Dunn JR. Primary care-based interventions to address the financial needs of patients experiencing poverty: a scoping review of the literature. *Int J Equity Health*. 2021;20(1):219. doi:10.1186/s12939-021-01546-8
42.	Peng Y, Hahn RA, Finnie RKC, et al. Permanent supportive housing with housing first to reduce homelessness and promote health among homeless populations with disability: a community guide systematic review. *J Public Health Manag Pract*. 2020;26(5):404-411. doi:10.1097/phh.0000000000001219. PMID: 32732712
43.	Ponka D, Agbata E, Kendall C, et al. The effectiveness of case management interventions for the homeless, vulnerably housed and persons with lived experience: a systematic review. *PLoS One*. 2020;15(4):e0230896. doi:10.1371/journal.pone.0230896. PMID: 32271769
44.	RAND Health Care*. Building the Evidence Base for Social Determinants of Health Interventions.* Office of the Assistant Secretary for Planning and Evaluation (ASPE), U.S. Department of Health & Human Services.
45.	Raphael JL, Rueda A, Lion KC, et al. The role of lay health workers in pediatric chronic disease: a systematic review. *Acad Pediatr*. 2013;13(5):408-420. doi:10.1016/j.acap.2013.04.015. PMID: 24011745
46.	Rasmussen B, Wynter K, Rawson HA, Skouteris H, Ivory N, Brumby SA. Self-management of diabetes and associated comorbidities in rural and remote communities: a scoping review. *Aust J Prim Health*. 2021;27(4):243-254. doi:10.1071/PY20110
47.	Rawal L, Sahle BW, Smith BJ, Kanda K, Owusu-Addo E, Renzaho AMN. Lifestyle interventions for type 2 diabetes management among migrants and ethnic minorities living in industrialized countries: a systematic review and meta-analyses. *BMJ Open Diabetes Res*. 2021;9(1):04. doi:10.1136/bmjdrc-2020-001924
48.	Reeves TJ, Mathis TJ, Bauer HE, et al. Racial and ethnic disparities in health outcomes among long-term survivors of childhood cancer: a scoping review. *Front*. 2021;9:741334. doi:10.3389/fpubh.2021.741334
49.	Ruiz Escobar EPS, Blanchard CM. Screening and referral care delivery services and unmet health-related social needs: a systematic review. *Prev Chronic Dis*. 2021;18:E78. doi:10.5888/pcd18.200569
50.	Seddighi H, Salmani I, Javadi MH, Seddighi S. Child abuse in natural disasters and conflicts: a systematic review. *Trauma Violence Abuse*. 2021;22(1):176-185. doi:10.1177/1524838019835973
51.	Smith SM, Wallace E, O'Dowd T, Fortin M. Interventions for improving outcomes in patients with multimorbidity in primary care and community settings*. Cochrane Database Syst Rev*. 2021;(1)1-111. doi:10.1002/14651858.CD006560.pub4
52.	Solomon EM, Wing H, Steiner JF, et al. Impact of transportation interventions on health care outcomes: a systematic review. *Med Care*. 2020;58(4):384-391. doi:10.1097/MLR.0000000000001292. PMID: 31985588
53.	Stormacq C, Wosinski J, Boillat E, Van den Broucke S. Effects of health literacy interventions on health-related outcomes in socioeconomically disadvantaged adults living in the community: a systematic review. *JBI Evid Synth*. 2020;18(7):1389-1469. doi:10.11124/JBISRIR-D-18-00023
54.	Taira BR, Kim K, Mody N. Hospital and health system-level interventions to improve care for limited English proficiency patients: a systematic review. *Jt Comm J Qual Patient Saf*. 2019;45(6):446-458. doi:10.1016/j.jcjq.2019.02.005. PMID: 30910471
55.	Thomas G, Lynch M, Spencer LH. A systematic review to examine the evidence in developing social prescribing interventions that apply a co-productive, co-designed approach to improve well-being outcomes in a community setting. *Int J Environ Res Public Health*. 2021;18(8):08. doi:10.3390/ijerph18083896
56.	Tsai C, Raphael S, Agnew C, McDonald G, Irving M. Health promotion interventions to improve oral health of adolescents: a systematic review and meta-analysis. *Community Dent Oral Epidemiol*. 2020;48(6):549-560. doi:10.1111/cdoe.12567
57.	van den Berk-Clark C, Doucette E, Rottnek F, et al. Do patient-centered medical homes improve health behaviors, outcomes, and experiences of low-income patients? A systematic review and meta-analysis. *Health Serv Res*. 2018;53(3):1777-1798. doi:10.1111/1475-6773.12737. PMID: 28670708
58.	Wouk K, Morgan I, Johnson J, et al. A systematic review of patient-, provider-, and health system-level predictors of postpartum health care use by people of color and low-income and/or uninsured populations in the United States. *J Womens Health*. 2021;30(8):1127-1159. doi:10.1089/jwh.2020.8738

© 2023 Cené CW et al. *JAMA Network Open.*

eTable 8. Inclusion and Exclusion Criteria for Scoping and Rapid Reviews*

Category	Inclusion	Exclusion
**Populations**	Demographic characteristics: General population, all ages, immigrants, racial/ethnic minorities.<br><br>For the review of the scoping review, eligible studies must report analyses addressing race or ethnicity<br><br>Health status: Pregnant women; studies targeting people with asthma, heart disease, diabetes, hypertension, mental health, or substance abuse; persons with multiple chronic conditions	For the review of the scoping review: No analyses addressing race or ethnicity<br><br>Health status: Studies targeting people with specific diseases other than asthma, heart disease, diabetes, hypertension, mental health, substance abuse
**Social needs**	Interventions addressing individual social needs<br><br>Food insecurity, housing instability and quality, interpersonal violence (with the exclusion of intimate partner violence and child maltreatment†), education (including adult literacy and health literacy), financial strain, employment, social isolation, early childhood education and development, health care and primary care, transportation, utilities, legal services, childcare<br><br>Interventions targeting single or multiple domains; can address excluded domains, if 1 of the included domains above is addressed	Social needs addressed by US Preventive Services Task Force (USPSTF) recommendations: (depression,[1] unhealthy alcohol use,[2] healthy diet and physical activity,[3] drug use,[4] tobacco use,[5] intimate partner violence,[6] and child maltreatment[7]) or Centers for Disease Control and Prevention (CDC) (neighborhood and built environment[8])<br><br>Other social needs not included in Healthy People 2020
**Interventions**	Individual level (eg, referral to social services, provision of information about resources)<br><br>Health care system level (eg, policies, programs, staff training, primary care collaboration with community services)<br><br>Adjustment interventions or assistance interventions	Public health/community-level policies<br><br>Individual-level interventions that target medical conditions/needs alone (rather than social needs alone or social needs in combination with medical needs)<br><br>Advocacy, alignment, or awareness interventions
**Comparisons**	Contemporaneous or historical comparator (usual care or wait-list controls)	No comparator
**Outcomes**	Behavioral outcomes, health outcomes, health care utilization outcomes, harms/unanticipated outcomes	Process outcomes, social needs outcomes, cost outcomes, provider outcomes
**Timing**	All	None
**Setting**	Any setting linked with the health care system; conducted in the United States	Conducted outside the United States; no link with US health care system
**Study design**	Randomized clinical trials, nonrandomized controlled trials, cohort studies, case-control studies (cases and controls defined by presence or absence of outcome), single-arm studies with data collected before and after the intervention (pre-intervention-post-intervention)<br><br>Studies of head-to-head comparisons (ie, comparative effectiveness studies) treated as pre-post interventions for each arm	Case series, case reports, dissertations, modeling studies, screening tool validation studies, studies with a comparison group defined by the absence of social needs
**Language**	English	Non-English

* Unless otherwise specified, these criteria were first specified in PCORI's scoping review and evidence map.
** We excluded child maltreatment and intimate partner violence from interpersonal violence because these topics were covered by the USPSTF.

© 2023 Cené CW et al. *JAMA Network Open.*

Abbreviations: CDC=Centers for Disease Control and Prevention; US=United States; USPSTF=U.S. Preventive Services Task Force.

eFigure 1. Screening Approach for PCORI's Scoping Review and Evidence Map

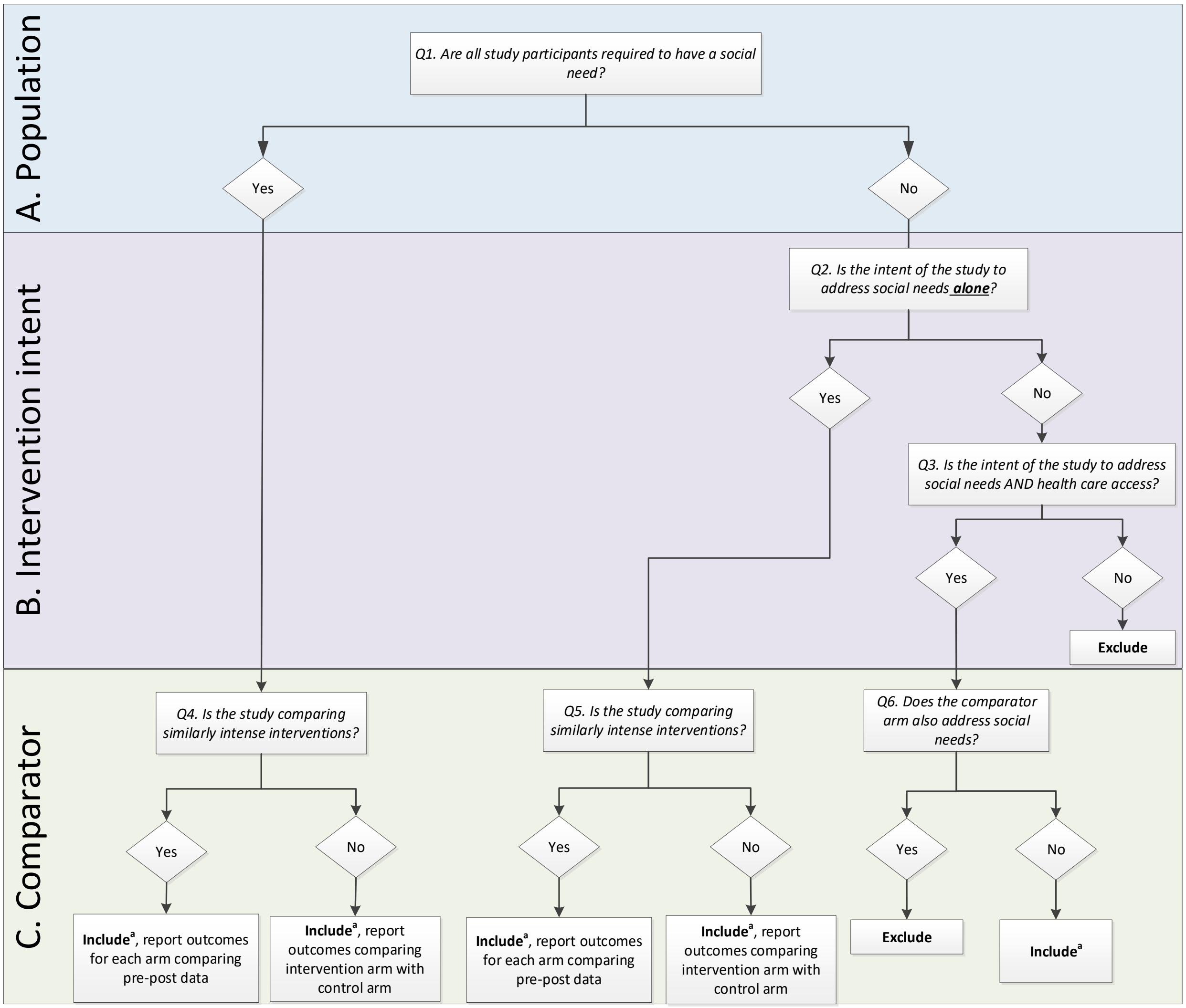

[a]if study meets all other eligibility criteria.

© 2023 Cené CW et al. *JAMA Network Open.*

eAppendix 1. Disposition of Studies Identified

Among the 157 studies included in the scoping review, 152 were among multiracial or multiethnic populations, therefore meeting our inclusion criteria for this review (eFigure 2).

eFigure 2. Articles Included and Excluded for the Social Needs and Racial Health Equity Review of a Scoping Review

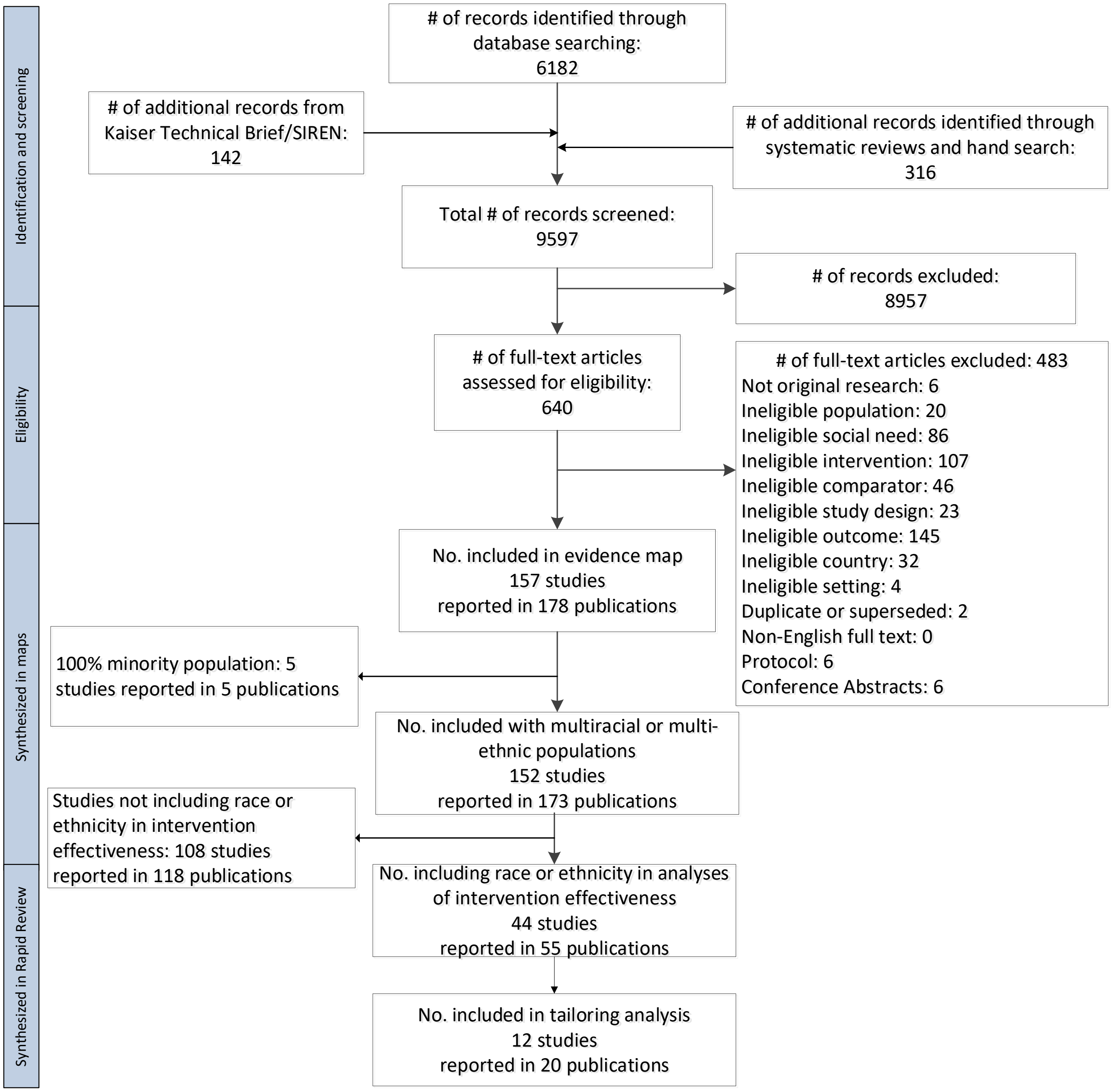

eAppendix 2. Risk-of-Bias Assessment

PCORI's scoping review and evidence map[9] categorized studies first by study design: randomized controlled trial (RCT) cohort studies with comparison (including controlled clinical

© 2023 Cené CW et al. *JAMA Network Open.*

trials, retrospective cohort studies, and prospective cohort studies); case-control, single-arm studies reporting data before and after the intervention; and other nonrandomized studies. We assessed studies with external controls using the Cochrane risk-of-bias (ROB-2.0) instrument for trials[10] or ROBINS-I[11] for observational studies or nonrandomized experimental studies. Single-arm studies with data before and after the intervention inherently have limited ability to assert causal inference when compared with studies with external controls; thus, we did not rate the risk of bias of these studies but relied on study strategy to infer that causal inference cannot be made.

We did not rate the risk of bias of comparative effectiveness studies that we treated as single-arm studies; they, particularly if randomized, would have had inherent features that would have protected against regression to the mean and confounding. Using these studies as individual single-arm studies does not imply that their risk of bias was inherently high. One reviewer evaluated the risk of bias for each study; a second spot-checked ratings for quality. Differences were resolved through discussion.

© 2023 Cené CW et al. *JAMA Network Open.*

eTable 9. Individual Study Quality Assessment of Randomized Controlled Trials Based on Cochrane RoB 2.0

Author, Year (Reference # in Main Paper)	Risk of Bias Arising From the Randomization Process	Risk of Bias Due to Deviations From the Intended Interventions	Missing Outcome Data	Risk of Bias in Measurement of the Outcome	Risk of Bias in Selection of the Reported Result	Overall Risk of Bias
Berkowitz et al, 2019[12] (135)	Low	Low	Some concerns	Low	Low	Medium
Birkhead et al, 1995[13] (163)	Some concerns	Some concerns	Some concerns	Some concerns	Some concerns	Medium
Duncan et al, 2020[46] (170)	Low	Low	Low	Low	Low	Low
Gottlieb et al, 2016[53] (25)	Low	Low	Some concerns	Low	Low	Medium
Guevara et al, 2020[52] (129)	Low	Low	Some concerns	Low	Low	Medium
Hilgeman et al, 2014[14] (128)	Low	Low	Low	Low	Low	Low
Horwitz et al, 2005[15] (82)	Low	Low	Low	Low	Low	Low
Kelley et al, 2020[16] (68)	Low	Low	Low	Low	Low	Low
Krieger et al, 1999[17] (96)	Low	Low	High	Low	Low	High
Krieger et al, 2009[18] (87)	Low	Low	Some concerns	Some concerns		Medium
Krieger et al, 2005 (28)	Low	Low	High	Low	Low	High
Krieger et al, 2015[19] (95)	Low	Low	Low	Some concerns	Low	Medium
Liss et al, 2019[20] (58)	Some concerns	Some concerns	Low	Low	Low	Medium
Melnikow et al, 1997[21] (94)	Low	Low	Low	Low	Low	Low
Nyamathi et al, 2001[22] (102)	Some concerns	Low	High	Some concerns	Low	High
Tomita 2012[23] (35)	Low	Low	High	Low	Low	High
Towfighi et al, 2021[24] (161)	Low	Low	Low	Low	Low	Low

© 2023 Cené CW et al. *JAMA Network Open.*

eTable 10. Individual Study Quality Assessment of Nonrandomized Studies of Interventions Using ROBINS-I*

Author, Year (Reference # in Main Paper)	Confounding	Selection	Classification	Deviations From Intended Inter-ventions	Missing Data	Measurement of Outcomes	Selection of Reported Result	Overall Risk-of-Bias Judgment
Chaiyachati et al, 2018[25] (92)	Moderate	Low	Low	Low	Low	Low	Low	Medium
Chaiyachati et al, 2018[26] (93)	Low	Low	Low	Moderate	Low	Low	Low	Medium
Ciaranello et al, 2006[27] (85)	Moderate	Low	Low	Low	Low	Moderate	Low	Medium
Duru et al, 2020[28] (66)	Moderate	Low	Low	Moderate	Low	Low	Low	Medium
Foster et al, 2018[29] (151)	Serious[†]							High
Gusmano et al, 2018[30] (63)	Moderate	Low	Low	Moderate	Low	Low	Low	Medium
Lindau et al, 2019[31] (74)	Low	Low	Low	Low	Low	Moderate	Low	Medium
Mendelsohn et al, 2001[32] (29)	Moderate	Low	Low	Low	Moderate	Moderate	Low	Medium
Morales et al, 2016[33] (30)	Moderate	Low	Low	Low	Low	Low	Low	Medium
Moreno et al, 2021[54] (168)	Moderate	Low	Low	Low	Low	Low	Low	Medium
Shah et al, 2011[50] (83)	Moderate	Low	Low	Low	Low	Low	Low	Medium
Tessaro et al, 1997[34] (47)	Serious[†]							High

© 2023 Cené CW et al. *JAMA Network Open.*

Author, Year<br><br>(Reference # in Main Paper)	Confounding	Selection	Classification	Deviations From Intended Inter-ventions	Missing Data	Measurement of Outcomes	Selection of Reported Result	Overall Risk-of-Bias Judgment
Tsai and Rosenheck, 2012[35] (143)	Moderate	Low	Low	Low	Low	Low	Low	Moderate

[*] Risk Of Bias In Non-randomized Studies - of Interventions.
[†] We did not rate subsequent domains if confounding was rated as serious, because a serious rating for confounding would lead to an overall serious rating.

© 2023 Cené CW et al. *JAMA Network Open.*

eTable 11. Key Characteristics of Studies That Included Race or Ethnicity in Their Analyses

Study Characteristic	All Studies Including Race or Ethnicity in Analyses of Intervention Effectiveness	
	Studies (N = 44)/Interventions (N = 49)	
	n	%
**Study Design**		
Randomized controlled trial	16	36.4
Cohort with comparison	13	29.5
Single-arm study comparing data before and after intervention	13	29.5
Comparative effectiveness	2	4.5
Case-control	0	0.0
**Quality**		
High	6	13.6
Medium	18	40.9
Low	5	11.4
Not rated	15	34.1
**Age Group**		
Children (<18 years) or children and their families	8	18.2
Adolescents/young adults (eg, 13-20 years)	4	9.1
Adults (≥18 years)	34	77.3
Older adults (eg, ≥50 years)	31	70.5
Only older adults (eg, ≥50 years)	1	2.3
**Majority Race or Ethnicity***		
Majority Black/non-Hispanic Black	11	25.0
Majority White/non-Hispanic White	9	20.5
Majority Hispanic/Latino	6	13.6
Majority Asian/Pacific Islander	1	2.3
Majority Native American/American Indian/Indigenous	0	0.0
Other (Other than Hispanic, White, Black, Asian)	1	2.3
No single group was a majority	15	34.1
Not reported	1	2.3
**Social Needs Addressed**		
Childcare assistance	0	0.0
Early childhood education and development access and quality	3	6.8
Education access and quality	6	13.6
Employment assistance	8	18.2
Financial strain assistance	6	13.6
Food security assistance	14	31.8
Health care services access and quality	30	68.2
Housing stability and quality	19	43.2
Interpersonal violence assistance	0	0.0
Legal services assistance	5	11.4
Social isolation assistance	4	9.1
Transportation assistance	15	34.1
Utilities assistance	1	2.3
Additional unspecified domains addressed	19	43.2
Multidomain intervention (none of the above)	1	2.3
**Intervention Components†**		

© 2023 Cené CW et al. *JAMA Network Open.*

Study Characteristic	All Studies Including Race or Ethnicity in Analyses of Intervention Effectiveness	
	Studies (N = 44)/Interventions (N = 49)	
Screening	12	24.5
Patient education (including on health, other social need, or resources)	26	53.1
Health care provider education	3	6.1
Providing onsite resources	17	34.7
Passive referrals	15	30.6
Active assistance with resources (vouchers, appt scheduling, enrollment form help)	37	75.5
**Intervention Provider[†]**		
Health care providers (doctors, nurses, therapists, etc.)	14	28.6
Social worker	8	16.3
CHWs/navigators	17	34.7
Other nonprofessionals, including volunteers and study staff	24	49.0
Case manager	3	6.1
Not reported	2	4.1

[*] Majority defined as >50%.
[†] Reported by intervention.

Abbreviations: CHW=community health worker; n/N=number.

© 2023 Cené CW et al. *JAMA Network Open.*

eTable 12. Detailed Characteristics of Studies That Are Analytically Informative for Advancing Racial Health Equity Research (N=21)

Author, Year (Reference # in Main Paper) Study Design (Quality) Categorization Total N Participants	Intervention/ Intervention Setting Population Description Intervention Provider Tailoring Reported	Social Need(s) Addressed	Race/Ethnicity, n (%)	Overall Results	Results Reported by Race or Ethnicity
**Conceptually thoughtful for understanding root causes of racial health inequities and analytically informative for advancing racial health equity research**					
Krieger, 2005[36] (28)<br><br>Comparative effectiveness (Not rated)<br><br>N = 274	Home assessment and action plan with CHW follow-up to assist with completion and provision of resources to mitigate asthma/home-based care or single CHW visit and action plan with limited education/home-based care<br><br>Children with persistent asthma and their caregivers enrolled in Medicaid and living in King County (Washington)<br><br>CHWs/navigators<br><br>Tailoring: Yes	Housing stability and quality	No single group was a majority<br><br>Caregiver ethnicity<br>High intensity<br>Non-Hispanic White: (12.3)<br>Non-Hispanic African American: (31.9)<br>Vietnamese: (25.4)<br>Other Asian: (9.4)<br>Hispanic: (17.4)<br>Other: (3.6)<br><br>Low Intensity<br>Non-Hispanic White: (21.3)<br>Non-Hispanic African American: (27.9)<br>Vietnamese: (22.1)<br>Other Asian: (5.2)<br>Hispanic: (17.7)<br>Other: (5.9)	Mixed results for morbidity; positive effects for quality of life; positive effects for emergency departments and urgent care visits	No significant interactions between group allocation and caregiver's race/ethnicity for any of the primary outcomes (quality of life, urgent health care service use, or symptom days) (ie, the intervention effect was equivalent across caregivers of all racial/ethnic groups)
Szilagyi, 2002[37] (165)<br><br>Single arm[†] (Not rated)<br><br>N = 10 066	Lay outreach worker immunization tracking and promotion/primary care<br><br><br>Children ages 0-2 living in Monroe County (New York)	Transportation assistance<br>Health care services access and quality	Varied by region addressed<br><br>Inner city, %<br>Black (non-Hispanic): 58<br>Hispanic: 21	Positive effects for immunizations	Immunization rates at 12 months old, %<br>1996<br>White (non-Hispanic): 95<br>Black (non-Hispanic): 83<br>Hispanic: 84<br>All children: 90

© 2023 Cené CW et al. *JAMA Network Open.*

Author, Year (Reference # in Main Paper) Study Design (Quality) Categorization Total N Participants	Intervention/ Intervention Setting Population Description Intervention Provider Tailoring Reported	Social Need(s) Addressed	Race/Ethnicity, n (%)	Overall Results	Results Reported by Race or Ethnicity
Szilagyi, 2002 (continued)	Other nonprofessionals*<br><br>Tailoring: Yes		White (non-Hispanic): 15<br>Asian and others: 6<br><br>Rest of city, %<br>Black (non-Hispanic): 37<br>Hispanic: 15<br>White (non-Hispanic): 38<br>Asian and others: 10<br><br>Suburbs, %<br>Black (non-Hispanic): 7<br>Hispanic: 3<br>White (non-Hispanic): 84<br>Asian and others: 6<br><br>County, %<br>Black (non-Hispanic): 28<br>Hispanic: 10<br>White (non-Hispanic): 55<br>Asian and others: 7		1999<br>White (non-Hispanic): 94<br>Black (non-Hispanic): 86<br>Hispanic: 89<br>All children: 90<br><br>Disparity, %<br>1990<br>White-Black: 12 (*P* <.001)<br>White-Hispanic: 11 (*P* <.001)<br><br>1999<br>White-Black: 8 (*P* <.01)<br>White-Hispanic: 5 (*P* =.1)<br><br>Immunization rates at 24 months old, %<br>1996<br>White (non-Hispanic): 89<br>Black (non-Hispanic): 76<br>Hispanic: 74<br>All children: 83<br><br>1999<br>White (non-Hispanic): 88<br>Black (non-Hispanic): 81<br>Hispanic: 87<br>All children: 87<br><br>Disparity, %<br>1990<br>White-Black: 13 (*P* = 0.001)<br>White-Hispanic: 15 (*P* <.001)<br><br>1999

© 2023 Cené CW et al. *JAMA Network Open.*

Author, Year (Reference # in Main Paper) Study Design (Quality) Categorization Total N Participants	Intervention/ Intervention Setting Population Description Intervention Provider Tailoring Reported	Social Need(s) Addressed	Race/Ethnicity, n (%)	Overall Results	Results Reported by Race or Ethnicity
					White-Black: 7 (*P* =.4)<br>White-Hispanic: 1 (*P* = 0.7)
Towfighi, 2021[24] (161)<br><br>RCT (High)<br><br>N = 487	CHW-provided education and advanced practice clinician clinic visits and blood pressure monitors/primary care, telephone-based, home-based care<br>Adults (≥40 years) experiencing recent TIA, stroke, or ICH and high blood pressure<br><br>Health care providers, CHWs/navigators<br><br>Tailoring: Yes	Transportation assistance<br>Health care services access and quality<br>Social isolation assistance	Majority White/non-Hispanic White<br><br>Overall<br>White: 335 (70.4)<br>Black: 87 (18.3)<br>Asian: 30 (6.3)<br>≥1 Race: 10 (2.1)<br>Native American or Alaskan Native: 9 (1.9)<br>Native Hawaiian or other Pacific Islander: 5 (1.1)<br><br>Hispanic ethnicity: 347 (71.3)	No effects for mental health; no effects for functional status; no effects for quality of life; mixed results for other health outcomes (non-HDL, HbA1c, Log CRP, BMI); mixed results for diet; no effects for physical activity; no effects for other behavior (smoking); mixed results for frequency of health care use; mixed results for adherence to treatment	Changes in systolic blood pressure over time in usual care vs intervention, by subgroup<br>Systolic blood pressure, mmHg, mean (SD)<br>Hispanic<br>• Usual care at baseline: 147 (19)<br>• Usual care at 3 months: 137 (21)<br>• Usual care at 12 months:136 (21)<br>• Intervention at baseline: 145 (17)<br>• Intervention at 3 months: 134 (20)<br>• Intervention at 12 months: 133 (19)<br>• *P* value: 0.99<br>Not Hispanic<br>• Usual care at baseline: 142 (16)<br>• Usual care at 3 months: 134 (17)<br>• Usual care at 12 months:139 (24)<br>• Intervention at baseline: 140 (16)<br>• Intervention at 3 months: 134 (23)<br>• Intervention at 12 months: 132 (23)<br>• *P* value: 0.22

© 2023 Cené CW et al. *JAMA Network Open.*

Author, Year (Reference # in Main Paper) Study Design (Quality) Categorization Total N Participants	Intervention/ Intervention Setting Population Description Intervention Provider Tailoring Reported	Social Need(s) Addressed	Race/Ethnicity, n (%)	Overall Results	Results Reported by Race or Ethnicity
Towfighi, 2021 (continued)					Asian<br>• Usual care at baseline: 141 (14)<br>• Usual care at 3 months: 126 (18)<br>• Usual care at 12 months:131 (22)<br>• Intervention at baseline: 141 (20)<br>• Intervention at 3 months: 130 (23)<br>• Intervention at 12 months: 128 (15)<br>• *P* value: 0.68<br>Black<br>• Usual care at baseline: 142 (14)<br>• Usual care at 3 months: 135 (15)<br>• Usual care at 12 months:136 (22)<br>• Intervention at baseline: 141 (15)<br>• Intervention at 3 months: 137 (24)<br>• Intervention at 12 months: 136 (28)<br>• *P* value: 0.94<br>White<br>• Usual care at baseline: 148 (19)<br>• Usual care at 3 months: 139 (21)<br>• Usual care at 12 months:137 (23)

© 2023 Cené CW et al. *JAMA Network Open.*

Author, Year (Reference # in Main Paper) Study Design (Quality) Categorization Total N Participants	Intervention/ Intervention Setting Population Description Intervention Provider Tailoring Reported	Social Need(s) Addressed	Race/Ethnicity, n (%)	Overall Results	Results Reported by Race or Ethnicity
Towfighi, 2021 (continued)					- Intervention at baseline: 144 (18) - Intervention at 3 months: 133 (19) - Intervention at 12 months: 133 (19) *P* value: 0.64 Other - Usual care at baseline: 142 (24) - Usual care at 3 months: 122 (10) - Usual care at 12 months:138 (20) - Intervention at baseline: 141 (17) - Intervention at 3 months: 139 (19) - Intervention at 12 months: 140 (20) *P* value: 0.14 (ie, no improvements in BP control compared with usual case) Other potential moderators, including site, race, ethnicity, and preferred language, were not associated with primary or secondary outcomes
**Not conceptually thoughtful for understanding root causes of racial health inequities but analytically informative for advancing racial health equity research**					
Chaiyachati, 2018[26]<br>(93)	Free transportation to medical appointment/other	Transportation assistance	Majority Black/non-Hispanic Black<br><br>Intervention<br>White: 10 (2.5)	No effects for emergency departments and urgent care visits; no effects for clinic	No statistically significant intervention results analyses by race/ethnicity for missed appointments, same-day cancellation, and no show

© 2023 Cené CW et al. *JAMA Network Open.*

Author, Year (Reference # in Main Paper) Study Design (Quality) Categorization Total N Participants	Intervention/ Intervention Setting Population Description Intervention Provider Tailoring Reported	Social Need(s) Addressed	Race/Ethnicity, n (%)	Overall Results	Results Reported by Race or Ethnicity
Cohort with comparison (Medium)<br>N = 786<br><br>Chaiyachati, 2018 (continued)	Adults receiving Medicaid and living in high-poverty neighborhood<br>Other nonprofessionals*<br>Tailoring: No		Black: 371 (94.2)<br>Other/mixed: 13 (3.3)<br>Hispanic: 2 (0.5)<br>Non-Hispanic: 392 (99.5)<br><br>Control<br>White: 4 (1.0)<br>Black: 377 (96.2)<br>Other/mixed: 11 (2.8)<br>Hispanic: 1 (0.3)<br>Non-Hispanic: 391 (99.7)	attendance; positive effects for missed appointments;	Results by race (not including intervention effect)<br>All missed appointments, OR (95% CI)<br>• Black: 0.94 (0.70 to 1.26), *P* =.66<br>• Non-Black: 3.86 (0.59 to 25.3), *P* = .16<br>Ethnicity<br>• Hispanic: NR<br>• Non-Hispanic: 0.99 (0.74 to 1.32), *P* = .92<br>Same-day cancellation, OR (95% CI)<br>• Black: 0.87 (0.55 to 1.36), *P* = .56<br>• Non-Black: 0.83 (0.05 to 15.1), *P* = .90<br>Ethnicity<br>• Hispanic: NR<br>• Non-Hispanic: 0.85 (0.55 to 1.33), *P* = .49<br>No show, OR (95% CI)<br>• Black: 0.99 (0.71 to 1.38), *P* = .97<br>• Non-Black: 6.25 (0.60 to 64.9), *P* = .13<br>Ethnicity<br>• Hispanic: NR

© 2023 Cené CW et al. *JAMA Network Open.*

Author, Year (Reference # in Main Paper) Study Design (Quality) Categorization Total N Participants	Intervention/ Intervention Setting Population Description Intervention Provider Tailoring Reported	Social Need(s) Addressed	Race/Ethnicity, n (%)	Overall Results	Results Reported by Race or Ethnicity
Chaiyachati, 2018 (continued)					• Non-Hispanic: 1.07 (0.78 to 1.48), *P* =.70
Chan, 2009[38] (126)<br><br>Single arm[†] (Not rated)<br><br>N = 725<br><br>Chan, 2009 (continued)	Computerized referral system to community clinics/ED, primary care, web-based care<br><br>People without primary care providers visiting an ED<br><br>Health care providers	Health care services access and quality	NR	Positive effects for emergency departments and urgent care visits; positive effects for post-discharge primary care visits	For the multivariate logistic regression analysis to identify which factors were associated with adherence with follow-up at the community clinics during the post period, there were no independent associations among patient characteristics (age, sex, race/ethnicity, marital status, ED visit acuity, and health coverage insurance status) and period
Duncan 2020[39] (170)<br><br>RCT (High)<br><br>N = 5882 (ITT analysis)	Telephone and clinic follow-up and individualized care plan including referral to community resources/outpatient clinic, telephone-based care<br><br>Adults with stroke or TIA discharged from hospital to home<br><br>Health care providers, Other nonprofessionals[*]<br><br>Tailoring: No	Food security assistance<br>Transportation assistance<br>Financial strain assistance<br>Health care services access and quality<br>Additional unspecified domains addressed	Majority White/non-Hispanic White<br><br>Intervention<br>White: 2112 (79.1)<br>Non-White: 559 (20.8)<br>Missing: 18 (0.67)<br><br>Usual Care<br>White: 2122 (67.2)<br>Non-White: 1037 (32.5)<br>Missing: 34 (1.1)<br><br>(Data for non-White calculated)	No effects for mortality; no effects for mental health; no effects for other health outcomes (general health); no effects for physical activity; no effects for other behavior (cognition); no effects for hospital readmissions; no effects for adherence to treatment; no effects for emergency departments and urgent care visits; no effects for other	Cognition, mean difference<br>Non-White: -0.96 (confidence limits: -1.80 to -0.11)<br>White: 0.04 (confidence limits: -0.57 to 0.65)<br>*P* = .09<br><br>Satisfaction with care coordination, mean difference<br>Non-White: 0.25 (confidence limits: 0.02 to 0.49)<br>White: 0.02 (confidence limits: -0.12 to 0.16)<br>*P* = .45<br>Stroke Impact Scale, White vs non-White: 9.73 (95% CI, 8.01 to 11.46), *P* < .0001<br><br>Regression models for ED

© 2023 Cené CW et al. *JAMA Network Open.*

Author, Year (Reference # in Main Paper) Study Design (Quality) Categorization Total N Participants	Intervention/ Intervention Setting Population Description Intervention Provider Tailoring Reported	Social Need(s) Addressed	Race/Ethnicity, n (%)	Overall Results	Results Reported by Race or Ethnicity
Duncan 2020 (continued)				health care use outcomes (risk of skilled nursing or rehab admission)	use, readmissions (all cause and stroke), mortality were adjusted for race<br>White race as predictor of skilled nursing or rehab admission: HR = 0.96 (2.5% CI = 0.72, 97.5% CI = 1.27), *P* = .765
Foster, 2018[29] (151)<br><br>Cohort with comparison (Low)<br><br>N = 85 701	Care coordination to support primary care appointments/ED, telephone-based care<br><br>Adults visiting an ED<br><br>Other nonprofessionals[*]<br>Tailoring: No	Health care services access and quality	No single group was a majority<br><br>Referred-successful linkage<br>African American: 646 (61)<br>Caucasian: 338 (31.9)<br>Other/not documented: 63 (5.9)<br>Hispanic: 6 (0.6)<br>Asian: 6 (0.6)<br>Referred-unsuccessful linkage<br>African American: 403 (64.1)<br>Caucasian: 187 (29.7)<br>Other/not documented: 33 (5.2)<br>Hispanic: 5 (0.8)<br>Asian: 1 (0.2)<br><br>Referred-assistance declined<br>African American: 262 (57.7)<br>Caucasian: 154 (33.9)<br>Other/not documented: 30 (6.6)	No effects for emergency departments and urgent care visits	Referred and successful linkage to primary care, n (%)<br>• African American: 646 (61.0)<br>• Caucasian: 338 (31.9)<br>• Other/not documented: 63 (5.9)<br>• Hispanic: 6 (0.6)<br>• Asian: 6 (0.6)<br>Referred and unsuccessful linkage to primary care, n (%)<br>• African American: 403 (64.1)<br>• Caucasian: 187 (29.7%)<br>• Other/not documented: 33 (5.2)<br>• Hispanic: 5 (0.8)<br>• Asian: 1 (0.2)<br>Referred and assistance declined, n (%)<br>• African American: 262 (57.7)<br>• Caucasian: 154 (33.9)

© 2023 Cené CW et al. *JAMA Network Open.*

Author, Year (Reference # in Main Paper) Study Design (Quality) Categorization Total N Participants	Intervention/ Intervention Setting Population Description Intervention Provider Tailoring Reported	Social Need(s) Addressed	Race/Ethnicity, n (%)	Overall Results	Results Reported by Race or Ethnicity
Foster, 2018 (continued)			Hispanic: 7 (1.5)<br>Asian: 1 (0.2)<br>Nonreferred<br>African American: 34 581 (41.3)<br>Caucasian: 39 386 (47.1)<br>Other/not documented: 8061 (9.6)<br>Hispanic: 1146 (1.4)<br>Asian: 463 (0.6)		• Other/not documented: 30 (6.6)<br>• Hispanic: 7 (1.5)<br>• Asian: 1 (0.2)<br>Nonreferred, n (%)<br>• African American: 34 581 (41.3)<br>• Caucasian: 39 386 (47.1)<br>• Other/not documented: 8061 (9.6)<br>• Hispanic: 1146 (1.4)<br>• Asian: 463 (0.6)<br>Among those referred to and accepting of care coordination, participants successfully and unsuccessfully linked to care, difference in proportions (95% CI)<br>White: -2.2 (-6.7, 2.4)
Glendenning-Napoli, 2012[40] (80)<br><br>Single arm† (Not rated)<br><br>N = 83	Case management/outpatient clinic, hospital, telephone and home-based<br><br>Patients with diabetes, hypertension, CHF, CAD and history of hospital admission or outpatient encounter<br><br>Social workers, CHWs/navigators<br><br>Tailoring: No	Food security assistance<br>Housing stability and quality<br>Financial strain assistance<br>Health care services access and quality	Majority White/non-Hispanic White<br><br>Non-Hispanic White: 43 (51.8)<br>Hispanic: 19 (22.9)<br>African American: 21 (25.3)	Positive effects for outpatient visits; positive effects for clinic attendance; positive effects for inpatient admissions	Acute outpatient encounters, pre-intervention mean (SD) vs post-intervention mean (SD)<br>Non-Hispanic White: 0.60 (0.93) vs 0.33 (0.71), *P* = .12<br>Hispanic: 0.84 (1.12) vs 0.11 (0.46), *P* = .02<br>African American: 0.76 (0.83) vs 0.29 (0.46), *P* = .01<br><br>Inpatient admissions, pre-intervention mean (SD) vs

© 2023 Cené CW et al. *JAMA Network Open.*

Author, Year (Reference # in Main Paper) Study Design (Quality) Categorization Total N Participants	Intervention/ Intervention Setting Population Description Intervention Provider Tailoring Reported	Social Need(s) Addressed	Race/Ethnicity, n (%)	Overall Results	Results Reported by Race or Ethnicity
Glendenning-Napoli, 2012 (continued)					post-intervention mean (SD), *P* value<br>Non-Hispanic White: 1.33 (1.13) vs 0.74 (1.16), *P* = .005<br>Hispanic: 1.16 (0.96) vs 0.32 (0.48), *P* = .0003<br>African American: 1.14 (1.15) vs 0.48 (0.98), *P* = .02<br><br>Clinic visits, pre-intervention mean (SD) vs post-intervention mean (SD), *P* value<br>Non-Hispanic White: 3.79 (4.39) vs 11.47 (9.53), *P* < .0001<br>Hispanic: 4.79 (3.44) vs 9.42 (5.94), *P* = .004<br>African American: 4.24 (3.75) vs 10.76 (10.62), *P* = .004<br><br>Cost of acute outpatient encounters, pre-intervention mean (SD) vs post-intervention mean (SD), *P* value<br>Non-Hispanic White: 1453 (2860) vs 941 (2829), *P* = .40<br>Hispanic: 2397 (3813) vs 331 (1441), *P* = .05<br>African American: 2090 (2852) vs 541 (1153), *P* = .04<br><br>Cost of inpatient admissions, pre-intervention mean (SD) vs post-intervention mean (SD), *P* value

© 2023 Cené CW et al. *JAMA Network Open.*

Author, Year (Reference # in Main Paper) Study Design (Quality) Categorization Total N Participants	Intervention/ Intervention Setting Population Description Intervention Provider Tailoring Reported	Social Need(s) Addressed	Race/Ethnicity, n (%)	Overall Results	Results Reported by Race or Ethnicity
Glendenning-Napoli, 2012 (continued)					Non-Hispanic White: 16 655 (22 158) vs 8483 (15 079), *P* = .01<br>Hispanic: 11 822 (27 911) vs 4616 (8880), *P* = .27<br>African American: 7931 (9431) vs 3449 (7709), *P* = .03<br>Cost of clinic visits, pre-intervention mean (SD) vs post-intervention mean (SD)<br>Non-Hispanic White: 941 (1143) vs 2277 (4751), *P* = .07<br>Hispanic: 1235 (1007) vs 1367 (981), *P* = .63<br>African American: 1052 (940) vs 4049 (8362), *P* = .12<br><br>Aggregate costs of health care utilization, pre-intervention mean (SD) vs post-intervention mean (SD), *P* value<br>Non-Hispanic White: 19 048 (22 254) vs 11 700 (15 994), *P* = .02<br>Hispanic: 15 454 (27 423) vs 6314 (9180), *P* = .16<br>African American: 11 073 (9573) vs 8039 (11 492), *P* = .25<br><br>All costs USD
Hilgeman, 2014[14] (128)	Veteran community outreach worker/home based	Health care services access and quality	Majority White/non-Hispanic White	Positive effects for clinic attendance; positive effects for	Logistical regression controlling for race, while predicting attendance at an

© 2023 Cené CW et al. *JAMA Network Open.*

Author, Year (Reference # in Main Paper) Study Design (Quality) Categorization Total N Participants	Intervention/ Intervention Setting Population Description Intervention Provider Tailoring Reported	Social Need(s) Addressed	Race/Ethnicity, n (%)	Overall Results	Results Reported by Race or Ethnicity
RCT (High)<br>Hilgeman, 2014 (continued)<br><br>N = 203	Rural veterans who had not accessed VA health care for ≥2 years<br><br>CHWs/navigators<br><br>Tailoring: No		Intervention<br>White: 52 (51.49)<br>Black: 49 (48.51)<br>Asian: 0 (0)<br>Hispanic: 0 (0)<br><br>Comparison<br>White: 67 (64.42)<br>Black: 34 (62.69)<br>Asian: 1 (0.96)<br>Hispanic: 2 (1.92)	other health care use outcomes (time to first clinical visit)	appointment within 6 months (yes/no), revealed no significant differences by race [Wald's χ2(1) = 0.63, *P* = .43; OR = 1.36, 95% CI, 0.69 to 2.68] or the race by group interaction.<br><br>Survival curves were presented for veterans by treatment group and separately by racial group to depict the significant group by race interaction.<br>General linear modeling was used to further explore the interaction detected in the Kaplan-Meier survival curve (F(1) = 10.61, *P* = .0014). Results indicated that regardless of race, veterans in the EEE [enhanced enrollment and engagement; treatment] group attended their first appointment at about 28 days (ie, White veterans M = 28.6, Black veterans M = 28.0, *P* = .97). For veterans in the AO [administrative outreach; control] group, time to attendance at an appointment was significantly different by race, such that Black veterans took twice as many days to attend an appointment as their White

© 2023 Cené CW et al. *JAMA Network Open.*

Author, Year (Reference # in Main Paper) Study Design (Quality) Categorization Total N Participants	Intervention/ Intervention Setting Population Description Intervention Provider Tailoring Reported	Social Need(s) Addressed	Race/Ethnicity, n (%)	Overall Results	Results Reported by Race or Ethnicity
Hilgeman, 2014 (continued)					counterparts (ie, M = 119.4 days vs M = 46.1 days, *P* < .0001, respectively.
Juillard, 2016[41] (26)<br><br>Single arm† (Not rated)<br><br>N = 459	Intensive case management/outpatient clinic<br><br>Patients presenting to emergency department with violent injury<br><br>CHWs/navigators<br><br>Tailoring: Yes	Housing stability and quality<br>Education access and quality<br>Employment assistance<br>Legal services<br>Additional unspecified domains	No single group was a majority<br><br>Black/African American: 215 (46.8)<br>Latino: 200 (43.5)<br>White: 23 (5.0)<br>Other (Native American, native Alaskan, native Hawaiian, Asian Pacific Islander, and mixed race): 21 (4.5)	Positive effects for other health outcomes (reinjury)	Reinjury, number of clients (%)<br>Black: No = 210 (98), Yes = 5 (2)<br>Latino: No = 178 (89), Yes = 22 (11)<br>White: No = 0 (0), Yes = 23 (100)<br>Other: No = 19 (68), Yes = 2 (7)<br>Unadjusted *P* < .001<br>χ2 measures of association showed no differences in intervention meeting client needs in terms of race, gender, or age
Kelley, 2020[16] (68)<br><br>RCT (High)<br><br>N = 100	Patient navigation/primary care, telephone-based care<br><br>Adults receiving Medicaid and visiting local ED 4-18 times in prior year<br><br>Health care providers, other nonprofessionals*<br><br>Tailoring: Yes	Food security<br>Housing stability and quality<br>Transportation assistance<br>Health care services access and quality<br>Additional unspecified domains	No single group was a majority<br><br>Intervention<br>White, non-Hispanic/Latino: 6 (12.24)<br>Black, non-Hispanic/Latino: 23 (46.94)<br>Hispanic/Latino: 19 (38.78)<br>Other: 1 (2.04)<br><br>Usual Care	No effects for outpatient visits; positive effects for emergency departments and urgent care visits; positive effects for inpatient admissions	Change in ED visits by race/ethnicity, reduced ED visits n (%) vs nonreduced ED visits n (%)<br>White, non-Hispanic/Latino: 3 (7.69) vs 2 (20.0)<br>Black, non-Hispanic/Latino: 18 (46.15) vs 5 (50.0)<br>Hispanic/Latino: 16 (41.03) vs 3 (30.0)<br>Other: 2 (5.13%) vs 0 (0)<br>*P* = .5789<br><br>No statistical differences between the groups in race/ethnicity among

© 2023 Cené CW et al. *JAMA Network Open.*

Author, Year (Reference # in Main Paper) Study Design (Quality) Categorization Total N Participants	Intervention/ Intervention Setting Population Description Intervention Provider Tailoring Reported	Social Need(s) Addressed	Race/Ethnicity, n (%)	Overall Results	Results Reported by Race or Ethnicity
Kelley,2020 (continued)			White, non-Hispanic/Latino: 12 (23.53)<br>Black, non-Hispanic/Latino: 25 (49.02)<br>Hispanic/Latino: 14 (27.45)<br>Other: 0		participants who reduced their ED utilization vs those who did not
Krieger, 1999[17] (96)<br><br>RCT (Low)<br><br>N = 241	Assistance with making appointments and removing barriers to care (childcare, etc.)/telephone-based care<br><br>Adults with high blood pressure and low income<br><br>CHWs/navigators<br><br>Tailoring: Yes	Health care services access and quality	Majority Black/non-Hispanic Black<br><br>Intervention<br>Black: (79.4)<br><br>Control<br>Black: (78.8)	Positive effects for other health care use outcomes (follow-up appointment with a medical care provider)	No significant (*P* < .05) interactions between intervention and age, sex, and race were present. The intervention thus appeared to be equally effective across ages, sexes, and races for appointment completion, although the sample size limited the study's ability to detect small differences in efficacy (<50% with 80% power) across subgroups
Krieger, 2009[18] (87)<br><br>RCT (Medium)<br><br>N= 309	CHW-delivered education and asthma mitigation support/home-based care<br><br>Children with persistent asthma and their caregivers enrolled in Medicaid and living in King County (Washington)<br><br>CHWs/navigators<br><br>Tailoring: Yes	Housing stability and quality<br>Health care services access and quality	No single group was a majority<br><br>Enrolled in study:<br>White: (11.3)<br>African American: (20.1)<br>Vietnamese: (11.0)<br>Other Asian: (5.8)<br>Hispanic: (47.9)<br>Other: (3.9)<br><br>Completed study:	Mixed results for functional status; positive effects for quality of life; mixed results for other health outcomes (asthma symptoms); positive effects for other behavior (environmental trigger reduction, self-medication management	In separate regression models for each of the 3 primary outcomes (caretaker quality of life, symptom-free days, and urgent health service use), no significant interactions between group allocation and child's age, baseline asthma severity, baseline symptom-free days, or caretaker's race/ethnicity and education

© 2023 Cené CW et al. *JAMA Network Open.*

Author, Year (Reference # in Main Paper) Study Design (Quality) Categorization Total N Participants	Intervention/ Intervention Setting Population Description Intervention Provider Tailoring Reported	Social Need(s) Addressed	Race/Ethnicity, n (%)	Overall Results	Results Reported by Race or Ethnicity
			White: (10.3)<br>African American: (20.3)<br>Vietnamese: (10.7)<br>Other Asian: (5.5)<br>Hispanic: (49.8)<br>Other: (3.3)	actions); no effects for emergency departments and urgent care visits; no effects for clinic attendance	Coefficients for regression models controlling for race/ethnicity were NR
Krieger, 2015[19] (95)<br><br>RCT (Medium)<br><br>N = 366	CHW-delivered education and asthma mitigation support/telephone-, home-, and web-based care<br><br>Adults with low household income and poorly controlled asthma living in King County (Washington)<br><br>Health care providers, CHWs/navigators<br><br>Tailoring: Yes	Housing stability and quality<br>Financial strain assistance<br>Education access and quality<br>Social isolation assistance<br>Legal services assistance<br>Health care services access and quality<br>Additional unspecified domains	No single group was a majority<br><br>Intervention<br>White: (26.0)<br>Black: (16.9)<br>Hispanic: (48.6)<br>Other: (8.5)<br><br>Control<br>White: (31.2)<br>Black: (16.4)<br>Hispanic: (45.0)<br>Other: (7.4)	Positive effects for mental health; no effects for functional status; positive effects for quality of life; positive effects for self-reported health status; positive effects for other health outcomes (asthma symptom-free days); no effects for frequency of health care use; no effects for emergency departments and urgent care visits	No significant interactions between race/ethnicity and study group for the 3 primary outcomes (symptom-free days, asthma-related quality of life, unscheduled health care use)
Lapham, 2008[42] (101)<br><br>Comparative effectiveness (Not rated)<br><br>N = 469	Case management and substance use counseling and peer-supervised housing OR peer-supervised housing and peer-resident support OR apartment or hotel housing only OR service referrals, bus fare, and payment for biweekly	Housing stability and quality<br>Health care services access and quality	No single group was a majority<br><br>Overall<br>Non-Hispanic White: (41)<br>Hispanic White (Hispanic): (31)	Mixed results for substance use	No statistically significant differences in substance use, housing stability, and employment status by race/ethnicity

© 2023 Cené CW et al. *JAMA Network Open.*

Author, Year (Reference # in Main Paper) Study Design (Quality) Categorization Total N Participants	Intervention/ Intervention Setting Population Description Intervention Provider Tailoring Reported	Social Need(s) Addressed	Race/Ethnicity, n (%)	Overall Results	Results Reported by Race or Ethnicity
	check-ins/<br>transitional housing, other<br><br>Homeless adults who abuse alcohol<br><br>Other nonprofessionals*<br><br>Tailoring: Yes		Native American: (18)<br>Other race groups: (10)		
Lyles, 2021[43] (159)<br><br>Single arm** (Not rated)<br><br>N = 179 (analyzed, 618 participants)	Peer mentor coaching/ telephone based<br><br>People with poorly controlled HbA1c or unknown control status<br><br>Other nonprofessionals*<br><br>Tailoring: Yes	Housing stability and quality<br>Transportation assistance<br>Health care services access and quality<br>Additional unspecified domains addressed	Majority Black/non-Hispanic Black<br>Black: 318 (51)<br>Hispanic/LatinX: 145 (23)<br>White: 35 (6)<br>Asian: 5 (1)<br>Other: 45 (7)<br>Missing/unknown: 70 (11)	Positive effects for other health outcomes (HbA1c)	Mean change in HbA1c by race/ethnicity<br>Black: -1.79%<br>Hispanic/Latinx: -1.51%<br>White: -1.36%
Martinez, 2006[44] (117)<br><br>Single arm[†,‡] (Not rated)<br><br>N = 236	Supportive housing with onsite services including case management, psychiatric care, health care, and vocational training/transitional housing<br><br>Formerly homeless, disabled, single adults with disabilities who entered supportive housing<br><br>Social workers, CHWs/navigators<br><br>Tailoring: No	Housing stability and quality<br>Employment assistance<br>Health care services access and quality	Majority Black/non-Hispanic Black<br><br>African American: 126 (53)<br>White: 76 (32)<br>Latino: 18 (8)<br>Native American: 11 (5)<br>Asian: 5 (2)	Positive effects for emergency departments and urgent care visits; positive effects for inpatient admissions	Coefficients for White, Latino, Asian and Native American (African American = reference group) were NS in the case-control model of predictors of change in the number of emergency department visits from year 1 to year 2

© 2023 Cené CW et al. *JAMA Network Open.*

Author, Year (Reference # in Main Paper) Study Design (Quality) Categorization Total N Participants	Intervention/ Intervention Setting Population Description Intervention Provider Tailoring Reported	Social Need(s) Addressed	Race/Ethnicity, n (%)	Overall Results	Results Reported by Race or Ethnicity
Mendelsohn, 2001[32]<br>(29)<br><br>Cohort with comparison<br>(Medium)<br><br>Mendelsohn, 2001 (continued)<br><br>N = 138	Literacy support program based on Reach Out and Read/primary care<br><br>Children ages 2-5.9 years old and of Latino or Black ethnicity<br><br>Health care providers, other nonprofessionals*<br><br>Tailoring: No	Early childhood education and development access and quality	Majority Hispanic/ Latino<br><br>Intervention (Families)<br>Latino: 79.6%<br>Black: 20.4%<br><br>Comparison (Families)<br>Latino: 64.4%<br>Black: 35.6%	Mixed results for child development	Unadjusted analysis of Latino families (n = 86), difference in score between intervention and comparison clinic<br>Receptive vocabulary score: 10.5 points (95% CI, 4.8, 16.3; t = 3.7; *P* <.001)<br>Expressive vocabulary score, difference in score: 5.3 points (95% CI, 0.3, 10.3; t = 2.1; *P* = .04)<br><br>Unadjusted analysis of all families (Black and Latino, n = 138), difference in score between intervention and comparison clinic<br>Receptive vocabulary score: 9.7 points (95% CI, 4.5 15.0; t=3.7; *P* <.001)<br>Expressive vocabulary score: 2.7 points (95% CI, -1.7, 7.1; t = 1.2; *P* = .23)<br><br>Multiple regression where main predictor is "child attends intervention clinic" (Latino ethnicity as a covariate [Black is reference])<br>For receptive language score<br>• Latino ethnicity: B = 4.6, *P* = .15<br>For expressive language score<br>Latino ethnicity:<br>B = -0.5, *P* = .83

© 2023 Cené CW et al. *JAMA Network Open.*

Author, Year (Reference # in Main Paper) Study Design (Quality) Categorization Total N Participants	Intervention/ Intervention Setting Population Description Intervention Provider Tailoring Reported	Social Need(s) Addressed	Race/Ethnicity, n (%)	Overall Results	Results Reported by Race or Ethnicity
Slesnick, 2007[45] (62)<br><br>Single arm (Not rated)[†]<br><br>N = 172<br><br>Slesnick, 2007 (continued)	Case management and individual therapy/homeless center<br><br>Adolescents and young adults experiencing homelessness<br>Other nonprofessionals[*]<br><br>Tailoring: No	Housing stability and quality<br>Education access and quality<br>Employment assistance<br>Health care services access and quality<br>Additional unspecified domains	No single group was a majority<br><br>White (37.2)<br>Hispanic (31.4)<br>Native American (12.2)<br>African American or Black (7.6)<br>Mixed ethnicity (11.6)	Positive effects for mental health; positive effects for substance use; positive effects for clinic attendance	Individual characteristics including age, education level, and ethnicity were not predictive of change in homelessness<br><br>Coefficients for ethnicity were all NS for change in alcohol and drug use, change in percent days housed, or change in psychological distress (all *P* > .05).<br><br>Coefficients for ethnicity for likelihood of being employed, being in school, and having access to medical care were NR
Tessaro, 1997[34] (47)<br><br>Cohort with comparison (Low)<br><br>N = 14 714	Lay health worker pregnancy health promotion/home based<br><br>Pregnant people at risk for poor birth outcomes<br><br>CHWs/navigators<br><br>Tailoring: No	Housing stability and quality<br>Education access and quality<br>Employment assistance<br>Early childhood education and development access and quality<br>Health care services access and quality<br>Additional unspecified domains	Majority Black/non-Hispanic Black<br><br>Maternal outreach worker program<br>African American: (61.8)<br>Caucasian: (38.2)<br><br>Care coordination program<br>African American: (59.4)<br>Caucasian: (40.6)	No effects for low birth weight; no effects for preventive care utilization; mixed results for prenatal visits	Adequacy of prenatal care among participants and comparisons, %<br>African American (live births: 893 among participants, 5607 among comparisons)<br>• Adequate: Participants 60.7%, Comparisons 63.8%<br>• Intermediate: Participants 32.6%, Comparisons 31.5%<br>• Inadequate: Participants 6.7%, Comparisons 4.7% (row mean score, *P* < .05)

© 2023 Cené CW et al. *JAMA Network Open.*

Author, Year (Reference # in Main Paper) Study Design (Quality) Categorization Total N Participants	Intervention/ Intervention Setting Population Description Intervention Provider Tailoring Reported	Social Need(s) Addressed	Race/Ethnicity, n (%)	Overall Results	Results Reported by Race or Ethnicity
Tessaro, 1997 (continued)					Caucasian (live births: 724 among participants, 7120 among comparisons)<br>Adequate: Participants 77.4%, Comparisons 75.1%<br>Intermediate: Participants 19.7%, Comparisons 22.8%<br>Inadequate: Participants 2.9%, Comparisons 2.1%<br>(row mean score, *P* > .05 [no statistically significant difference in distribution])<br>Adverse events among participants, observed vs expected number African American (total births = 895)<br>Low birth weight: Observed 104, Expected 117; Difference -13 (*P* = .12)<br>Very low birth weight: Observed 14, Expected 20; Difference -6 (*P* = .10)<br>Caucasian (total births = 724):<br>• Low birth weight: Observed 62, Expected 61; Difference 1 (*P* = .58)<br>• Very low birth weight: Observed 7, Expected 7; Difference 0 (*P* = .60)<br>Caucasian people were significantly more likely to

© 2023 Cené CW et al. *JAMA Network Open.*

Author, Year (Reference # in Main Paper) Study Design (Quality) Categorization Total N Participants	Intervention/ Intervention Setting Population Description Intervention Provider Tailoring Reported	Social Need(s) Addressed	Race/Ethnicity, n (%)	Overall Results	Results Reported by Race or Ethnicity
Tessaro, 1997 (continued)					report a high number of emotional (*P* < .01) and informational (*P* = .001) needs than African American people. The assistance needs for African American people and Caucasian people were similar.<br><br>A significantly higher percentage of African American people reported a high level of emotional (*P* < .05) and assistance (*P* < .01) needs met compared with Caucasian people, regardless of participant/comparison status. No difference in information needs met associated with race.
Whorms, 2021[46] (171)<br><br>Single arm† (Not rated)<br><br>N = 15 577	Rideshare service/outpatient clinic<br><br>Patients scheduling for imaging appointment and experiencing transportation difficulties<br><br>Other nonprofessionals*<br><br>Tailoring: No	Transportation assistance	Majority White/non-Hispanic White<br><br>Rideshare appointments<br>White: 114<br>Black/African American: 11<br>Asian: 8<br>Hispanic: 12<br>Other: 3<br>Nonrideshare appointments, pre-intervention<br>White: 6041<br>Black/African American: 383	No effects for missed appointments; positive effects for other health care use outcomes (being on time for appointments)	Missed appointments, non-White participants, n (%)<br>Pre-intervention: 323 (31.6)<br>Post-intervention: 288 (30.6)<br>Adjusted OR: 1.19 (95% CI, 0.77 to 1.84), *P* = .429<br><br>Analyses adjusted for race as a potential confounder

© 2023 Cené CW et al. *JAMA Network Open.*

Author, Year (Reference # in Main Paper) Study Design (Quality) Categorization Total N Participants	Intervention/ Intervention Setting Population Description Intervention Provider Tailoring Reported	Social Need(s) Addressed	Race/Ethnicity, n (%)	Overall Results	Results Reported by Race or Ethnicity
Whorms, 2021 (continued)			Asian: 357<br>Hispanic: 749<br>Other: 491<br>Nonrideshare appointments, post-intervention<br>White: 5769<br>Black/African American: 353<br>Asian: 277<br>Hispanic: 720<br>Other: 215		
Xiang, 2019[47] (78)<br><br>Single arm† (Not rated)<br><br>N = 586	Care coordination and case management/hospital, telephone-based care<br><br>Adults with ≥5 hospital admissions in prior year<br><br>Social workers<br>Tailoring: No	Health care services access and quality<br>Additional unspecified domains	Majority Black/non-Hispanic Black<br><br>White: (39.8)<br>African American: (52.7)<br>Other: (7.5)	Positive effects for hospital readmissions; positive effects for emergency departments and urgent care visits; positive effects for inpatient admissions; no effects for hospital days	In multiple regression models, race was not associated with changes in health services utilization and cost measures after the intervention; results from regression were NR

* Other nonprofessionals include nonclinicians such as CHWs, lay workers, volunteers.

† Pre-intervention to post-intervention changes or changes over time serve as the proxy for the intervention effect in single-arm studies.

‡ Study has a randomized subanalysis.

Abbreviations: AO=administrative outreach; BMI=body mass index; BP=blood pressure; CAD=coronary artery disease; CHF=congestive heart failure; CHW=community health worker; CI=confidence interval; CRP=C-reactive protein ; ED=emergency department; EEE=enhanced enrollment and engagement; HDL=high-density lipoprotein; HR=hazard ratio; ICH=intracerebral hemorrhage ; ITT=intention to treat; N=number; NR=not reported; NS=not significant ; OR=odds ratio; RCT=randomized controlled trial; SD=standard deviation; TIA=transient ischemic attack ; USD=US dollar; VA=Veterans Affairs.

© 2023 Cené CW et al. *JAMA Network Open.*

eTable 13. Detailed Characteristics of Studies with Analyses That Are Not Informative for Advancing Racial Health Equity Research (N = 23)

Author, Year (Reference # in Main Paper)<br><br>Study Design (Quality)<br>Reported N	Intervention/ Intervention Setting<br>Population Description<br>Intervention Provider<br>Tailoring Reported	Social Need(s) Addressed	Majority Race or Ethnicity	Overall Results and Direction of Effect	Information About Race or Ethnicity (Unrelated to Intervention Effects)
**Conceptually thoughtful for understanding root causes of racial health inequities but not analytically informative for advancing racial health equity research**					
Crisanti, 2017[48]<br>(142)<br><br>Single arm*<br>(Not rated)<br><br>N = 237	Housing and support services delivered by peer support worker/outpatient clinic, home-based care<br><br>Homeless adults or adults at risk for homelessness, diagnosed with serious mental illness or substance use disorder<br><br>Case manager<br><br>Tailoring: No	Housing stability and quality<br>Education access and quality<br>Employment assistance<br>Legal services assistance	No single group was a majority	No effects for mental health; no effects for other health outcomes (overall health)	Psychological distress, difference of means (95% CI)<br>Hispanic: 1.0 (0.3 to 1.6)<br><br>Coefficient for race/ethnicity in the final model for overall health was NR
**Not analytically informative for advancing racial health equity research**					
Berkowitz, 2017[49]<br>(23)<br><br>Single arm*<br>(Not rated)<br><br>N = 1774	Assistance of advocate to prioritize unmet social needs, identify community resources, facilitate receipt of resource/primary care	Multiple domains	Majority White/non-Hispanic White	Mixed results for functional status	Race/ethnicity coefficients in regression models were NR

© 2023 Cené CW et al. *JAMA Network Open.*

Author, Year (Reference # in Main Paper)<br><br>Study Design (Quality) Reported N	Intervention/ Intervention Setting<br>Population Description<br>Intervention Provider<br>Tailoring Reported	Social Need(s) Addressed	Majority Race or Ethnicity	Overall Results and Direction of Effect	Information About Race or Ethnicity (Unrelated to Intervention Effects)
Berkowitz, 2017 (continued)	Adults screened for unmet social needs at internal medicine practices<br><br>Other nonprofessionals, including volunteers and study staff† Tailoring: No				
Berkowitz, 2019[12] (135)<br><br>RCT (Medium)<br><br>N = 122	Subsidized community supported agriculture vegetable share/primary care<br>Adults with a BMI >25 being treated at community health center<br><br>Other nonprofessionals, including volunteers and study staff†<br><br>Tailoring: No	Food security assistance	Majority White/non-Hispanic White	No effects for mental health; mixed results for functional status	Sensitivity analyses of Healthy Eating Index adjusted for race and other factors found similar benefit for the intervention (difference: 3.7, 95% CI, 0.3 to 7.0, *P* =.03)
Birkhead, 1995[13] (163)<br><br>RCT (Medium)<br><br>N = 459	Additional food vouchers as immunization incentive or passive referral for immunization/primary care, WIC clinic<br><br>Children (12 to 59 months) whose families presented for WIC certification	Health care access and quality<br>Food security assistance	Majority Hispanic/Latino	Positive effects for immunizations	Immunized during the intervention period, N (%)<br>Hispanic: 353 (75)<br>Black: 238 (73)<br>White and Asian: 27 (68)<br><br>Bivariate analysis of the relative risk of immunization, compared with White and Asian children<br>Hispanic: 1.12 (95% CI, 0.90 to 1.39)<br>Black: 1.06 (95% CI, 0.86-1.35);

© 2023 Cené CW et al. *JAMA Network Open.*

Author, Year (Reference # in Main Paper)<br><br>Study Design (Quality) Reported N	Intervention/ Intervention Setting<br>Population Description<br>Intervention Provider<br>Tailoring Reported	Social Need(s) Addressed	Majority Race or Ethnicity	Overall Results and Direction of Effect	Information About Race or Ethnicity (Unrelated to Intervention Effects)
	Other nonprofessionals, including volunteers and study staff†<br><br>Tailoring reported: No				not included in multivariate analyses that includes interventions
Chaiyachati, 2018[25]<br>(92)<br><br>Cohort with comparison (Medium)<br><br>N = 506	Free transportation to medical appointment/primary care<br><br>Adults with Medicaid scheduled for nonurgent primary care visit<br><br>Other nonprofessionals, including volunteers and study staff<br><br>Tailoring: No	Transportation assistance	Majority Black/non-Hispanic Black	Positive effects for clinic attendance	Race/ethnicity coefficients in the models of show rates for clinics were NR
Ciaranello, 2006[27]<br>(85)<br><br>Cohort with comparison (Medium)<br><br>N = 252	Integrated care by health care professionals and social workers/transitional housing, telephone-based<br><br>Adults living in transitional housing<br><br>Health care providers, social workers, other nonprofessionals, including volunteers and study staff†	Food security assistance<br>Housing stability and quality<br>Transportation assistance<br>Employment assistance<br>Health care services access and quality<br>Legal services assistance	No single group was a majority	No effects for mental health; positive effects for functional status; no effects for self-reported health status; positive effects for emergency departments and urgent care visits; no effects for inpatient admissions	Regression coefficients for race (non-White vs White) were NR

© 2023 Cené CW et al. *JAMA Network Open.*

Author, Year (Reference # in Main Paper)<br><br>Study Design (Quality) Reported N	Intervention/ Intervention Setting<br>Population Description<br>Intervention Provider<br>Tailoring Reported	Social Need(s) Addressed	Majority Race or Ethnicity	Overall Results and Direction of Effect	Information About Race or Ethnicity (Unrelated to Intervention Effects)
	Tailoring: No	Additional unspecified domains addressed			
Duru, 2020[28] (66)<br><br>Cohort with comparison (Medium)<br><br>N = 194 834	Care coordination, including links to social services and CHW-facilitated enrollment in services/NR<br>Adult Medicaid beneficiaries or United Healthcare beneficiaries with diabetes and supplemental Medicare or Medicare insurance or other supplemental<br><br>Health care providers, CHWs/navigators<br><br>Tailoring: No	Food security assistance<br>Health care services access and quality<br>Additional unspecified domains addressed	No single group was a majority	Mixed results for emergency departments and urgent care visits; mixed results for inpatient admissions	Coefficients for race were NR
Gottlieb, 2020[50] (167)<br><br>RCT (Medium)<br><br>N = 611	In-person navigation with provision of written resource information addressing participants' social needs or written information alone/urgent care, telephone-based care<br>Children (≤17 years of age) and caregiver residing in county of enrollment<br><br>CHWs/navigators<br><br>Tailoring: No	Food security assistance<br>Financial strain assistance<br>Any social need that arises in population addressed	Majority Hispanic/ Latino	No effects for quality of life; no effects for self-reported health status; no effects for other health outcomes (caregiver general health, perceived stress, or depression)	No statistically significant differences between groups in outcomes with or without adjustment for race or other factors

© 2023 Cené CW et al. *JAMA Network Open.*

Author, Year (Reference # in Main Paper)<br><br>Study Design (Quality) Reported<br>N	Intervention/ Intervention Setting<br>Population Description<br>Intervention Provider<br>Tailoring Reported	Social Need(s) Addressed	Majority Race or Ethnicity	Overall Results and Direction of Effect	Information About Race or Ethnicity (Unrelated to Intervention Effects)
Guevara, 2020[51](129)<br><br>RCT (Medium)<br><br>N = 120	Provision of books and reading promotion/primary care, home-based care, other (text messages)<br><br>Infants <30 days old at enrollment without neurodevelopmental disabilities or congenital malformations and receiving Medicaid<br><br>Health care providers, other nonprofessionals, including volunteers and study staff[†]<br><br>Tailoring: No	Early childhood education and development access and quality	Majority Black/non-Hispanic Black	No effects for child development	Coefficients for race were NR
Gusmano, 2018[30]<br>(63)<br><br>Cohort with comparison (Medium)<br><br>N = 17 195	Affordable housing with supportive services/home-based care<br><br>Residents of affordable housing, aged 65 years or older, enrolled in Medicare<br><br>Social workers<br><br>Tailoring: No	Food security assistance<br>Housing stability and quality<br>Transportation assistance<br>Social isolation assistance<br>Health care services access and quality<br>Additional unspecified domains addressed	Majority Asian/Pacific Islander	Positive effects for inpatient admissions; positive effects for hospital days; positive effects for other health care use outcomes	Coefficient (SE), odds ratio<br><br>Race/ethnicity (reference: White)<br>• Non-Hispanic Black: 0.737 (0.214), *P* = .001, OR = 2.090<br>• Hispanic: 0.697 (0.221), *P* =.002, OR=2.007<br>• Other: 0.115 (0.014), *P* =.001, OR = 1.1215<br>• Non-Hispanic Asian: 0.471 (0.145), *P* =.001, OR = 0.625
Horwitz, 2005[15]<br>(82)	Intensive case management/ emergency department	Health care services access and quality	No single group was a majority	No effects for emergency departments and urgent care visits; positive effects for	Intervention patients linked to primary care contacts, relative risk (95% CI):

© 2023 Cené CW et al. *JAMA Network Open.*

Author, Year (Reference # in Main Paper)<br><br>Study Design (Quality) Reported<br>N	Intervention/ Intervention Setting<br>Population Description<br>Intervention Provider<br>Tailoring Reported	Social Need(s) Addressed	Majority Race or Ethnicity	Overall Results and Direction of Effect	Information About Race or Ethnicity (Unrelated to Intervention Effects)
RCT (High)<br><br>N = 230	Uninsured adults not seen for only substance use or mental health issues<br><br>CHWs/navigators<br><br>Tailoring: No			post-discharge primary care visits; no effects for inpatient admissions	African American vs White: 0.80 (0.55 to 1.18)<br><br>Hispanic vs White: 1.07 (0.75 to 1.53)
Izumi, 2020[52] (169)<br><br>Single arm[*] (Not rated)<br><br>N = 48	Subsidized community supported agriculture share plus cooking education/outpatient clinic<br><br>Individuals receiving care at target clinic<br><br>CHWs/navigators, other nonprofessionals, including volunteers and study staff[†]<br>Tailoring: No	Food security assistance	Majority Hispanic/ Latino	Positive effects for mental health; positive effects for self-reported health status; mixed results for diet; mixed results for other behavior (fruit, vegetable, dark green vegetable, orange vegetable intake)	GEE models were adjusted for race/ethnicity, income, and number of shares picked up
Lindau, 2019[31] (74)<br><br>Cohort with comparison (Medium)<br><br>N = 420	Provision of list of community resources personalized to patient conditions/primary care, emergency department<br><br>Patients aged 45-74 who were beneficiaries of Medicare, Medicaid, or both who resided in the 16 zip-code study region	Food security assistance<br>Housing stability and quality<br>Transportation assistance<br>Utilities assistance<br>Education access and quality<br>Employment assistance<br>Health care services access and quality		No effects for quality of life	Adjusted SF-12 MCS, Estimate (SE)<br>Race/ethnicity: 3.82 (1.83), *P* = .04<br><br>Adjusted SF-12 PCS, Estimate (SE)<br>Race/ethnicity: -0.94 (0.84), *P* = .26<br><br>Adjusted logistic model for confidence in finding resources, Estimate (SE), 95% CI

© 2023 Cené CW et al. *JAMA Network Open.*

Author, Year (Reference # in Main Paper)<br><br>Study Design (Quality) Reported N	Intervention/ Intervention Setting<br>Population Description<br>Intervention Provider<br>Tailoring Reported	Social Need(s) Addressed	Majority Race or Ethnicity	Overall Results and Direction of Effect	Information About Race or Ethnicity (Unrelated to Intervention Effects)
	Health care providers, other nonprofessionals including volunteers and study staff†<br><br>Tailoring: No	Legal services assistance<br>Additional unspecified domains addressed			Race/ethnicity: 0.07 (0.35), 95% CI: -0.62, 0.76, *P* = .84<br><br>Adjusted logistic model for likelihood of recalling receiving intervention materials among intervention group participants, estimate, 95% CI Race/ethnicity: 0.27, (-1.39, 1.93), *P* = .75<br><br>Adjusted logistic model for conditional on recalling the likelihood of telling someone about the materials, estimate, 95% CI Race/ethnicity: 0.44, (-1.27, 2.15), *P* = .61
Liss, 2019[20] (58)<br><br>RCT (Medium)<br><br>N = 654	Transitional care/primary care, emergency department<br><br>Adults discharged from hospital or ED care and with no or inadequate usual source of care<br>Health care providers, social workers<br><br>Tailoring: No	Transportation assistance<br>Health care services access and quality<br>Additional unspecified domains addressed	No single group was a majority	No effects for mortality; no effects for self-reported health status; no effects for emergency departments and urgent care visits; mixed results for inpatient admissions; mixed results for other health care use outcomes (hospital encounters)	Race/ethnicity regression coefficients were NR
Martinez, 2006[44] (117)	Supportive housing with onsite services including case management, psychiatric care, health care,	Housing stability and quality<br>Employment assistance	Majority Black/non-Hispanic Black	Positive effects for emergency departments and urgent care visits; positive effects for inpatient admissions	Coefficients for White, Latino, Asian and Native American (African American = reference group) were NS in the case-control model of predictors of

© 2023 Cené CW et al. *JAMA Network Open.*

Author, Year (Reference # in Main Paper)<br><br>Study Design (Quality) Reported<br>N	Intervention/ Intervention Setting<br>Population Description<br>Intervention Provider<br>Tailoring Reported	Social Need(s) Addressed	Majority Race or Ethnicity	Overall Results and Direction of Effect	Information About Race or Ethnicity (Unrelated to Intervention Effects)
Single arm* (Not rated)<br><br>N = 236	and vocational training/transitional housing<br><br>Formerly homeless, disabled, single adults with disabilities who entered supportive housing, social workers, CHWs/navigators<br><br>Tailoring: No	Health care services access and quality			change in the number of emergency department visits from year 1 to year 2
Melnikow, 1997[21] (94)<br><br>RCT (High)<br><br>N = 104	Taxi voucher to prenatal care appointment/primary care<br><br>Pregnant individuals<br><br>Other nonprofessionals, including volunteers and study staff[†]<br><br>Tailoring: No	Transportation assistance, Health care services access and quality	Majority White/non-Hispanic White	Positive effects for prenatal visits	Controlling for ethnicity had no effect on the OR for appointment compliance among women who received a taxi voucher
Morales, 2016[33] (30)<br><br>Cohort with comparison (Medium)<br><br>N = 145	Assistance with food resources including SNAP or WIC enrollment/primary care<br><br>Pregnant individuals age 18 or older<br><br>NR<br>Tailoring: No	Food security assistance	Majority Hispanic/ Latino	Mixed results for functional status	Race/ethnicity coefficients in the regression models were NR

© 2023 Cené CW et al. *JAMA Network Open.*

Author, Year (Reference # in Main Paper)<br><br>Study Design (Quality) Reported<br>N	Intervention/ Intervention Setting<br>Population Description<br>Intervention Provider<br>Tailoring Reported	Social Need(s) Addressed	Majority Race or Ethnicity	Overall Results and Direction of Effect	Information About Race or Ethnicity (Unrelated to Intervention Effects)
Moreno, 2021[53] (168)<br><br>Cohort with comparison (Medium)<br><br>N = 1120	Social worker and CHW assessment of needs and assistance with connection to community resources and integration with primary care/primary care, telephone-based care, home-based care<br><br>Individuals requiring case management at level of intensity beyond that offered by medical group<br><br>Social workers, CHWs/navigators<br><br>Tailoring: No	Food security assistance<br>Housing stability and quality<br>Transportation assistance<br>Financial strain assistance<br>Health care services access and quality<br>Additional unspecified domains addressed	Other (other than Hispanic, White, Black, Asian)	Positive effects for emergency departments and urgent care visits; positive effects for inpatient admissions	Models were adjusted for race/ethnicity and other factors, but data are NR
Nyamathi, 2001[22] (102)<br><br>RCT (Low)<br><br>N = 845	Nurse and outreach worker-provided health education and assistance with local resources or peer mentor and outreach worker-provided education and assistance with local resources/NR<br><br>Homeless women (18-50 years of age) and their intimate partners (> age18)	Health care services access and quality<br>Additional unspecified domains addressed	Majority Black/non-Hispanic Black	Negative effects for mental health; no effects for substance use; no effects for other behavior (multiple sexual partners/sex without a condom)	Coefficients for race/ethnicity were NR

© 2023 Cené CW et al. *JAMA Network Open.*

Author, Year (Reference # in Main Paper)<br><br>Study Design (Quality) Reported<br>N	Intervention/ Intervention Setting<br>Population Description<br>Intervention Provider<br>Tailoring Reported	Social Need(s) Addressed	Majority Race or Ethnicity	Overall Results and Direction of Effect	Information About Race or Ethnicity (Unrelated to Intervention Effects)
	Health care providers, other nonprofessionals, including volunteers and study staff†<br><br>Tailoring: No				
Seligman, 2015[54]<br>(34)<br><br>Single arm*<br>(Not rated)<br><br>N = 687	Diabetes screening and diabetes-appropriate food distribution + primary care referral and self-management education/food pantry<br><br>Adult food pantry clients with an HbA1c≥6.5% or self-reported diabetes + presentation diabetes medication bottles<br><br>Other nonprofessionals, including volunteers and study staff†<br><br>Tailoring: No	Food security assistance<br>Health care services access and quality<br>Additional unspecified domains addressed	Majority Hispanic/ Latino	Positive effects for functional status; no effects for morbidity; positive effects for diet; positive effects for adherence to treatment	All regression models included race/ethnicity as a covariate. White race was the reference category, and race was not statistically significant in any model (all *P* >.05)
Shah, 2011[55]<br>(83)<br><br>Cohort with comparison<br>(Medium)<br><br>N = 258	Case management including linkage to community resources/primary care, outpatient clinic, hospital, telephone-based care, home-based care<br><br>Adults (18-64 years), with income below 200% of the federal poverty level,	Housing stability and quality<br>Transportation assistance<br>Health care services access and quality<br>Legal services assistance	No single group was a majority	Positive effects for emergency departments and urgent care visits; no effects for inpatient admissions; no effects for hospital days	Poisson regression results for number of ED visits (White reference group) estimate (standard error)<br>Asian: 0.5905 (0.3034), 0.0516<br>Black: -0.0565 (0.1295), 0.6625<br>Hispanic: 0.0474 (0.0864), 0.5836<br>Poisson regression results for number of inpatient admissions

© 2023 Cené CW et al. *JAMA Network Open.*

Author, Year (Reference # in Main Paper)<br><br>Study Design (Quality) Reported N	Intervention/ Intervention Setting<br>Population Description<br>Intervention Provider<br>Tailoring Reported	Social Need(s) Addressed	Majority Race or Ethnicity	Overall Results and Direction of Effect	Information About Race or Ethnicity (Unrelated to Intervention Effects)
Shah, 2011 (continued)	uninsured, and not eligible for any public insurance programs<br><br>Health care providers, CHWs/navigators<br><br>Tailoring: No	Additional unspecified domains addressed			(White reference group) estimate (standard error)<br>Black: 0.0886 (0.3159), 0.7791<br>Hispanic: -0.2656 (0.2249), 0.2376
Tomita, 2012[23] (35)<br><br>RCT (Low)<br><br>N = 150	Critical time Intervention including case worker assistance with community resources/home-based care<br><br>Individuals with psychotic disorders and history of homelessness living in transitional residences<br><br>Social workers<br><br>Tailoring: No	Housing stability and quality<br>Health care services access and quality<br>Additional unspecified domains addressed	Majority Black/non-Hispanic Black	Positive effects for hospital readmissions	Race/ethnicity coefficients in models were NR
Tsai, 2012[35] (143)<br><br>Cohort with comparison (Medium)<br><br>N = 31 246	Case management including support with housing vouchers and transition to community/NR<br><br>Homeless veterans with psychiatric, substance use, or general medical problems<br><br>Case manager<br><br>Tailoring: No	Housing stability and quality<br>Employment assistance<br>Financial strain assistance<br>Social isolation assistance	No single group was a majority	No effects for mental health; positive effects for quality of life; no effects for substance use	Coefficients for race/ethnicity were NR

© 2023 Cené CW et al. *JAMA Network Open.*

[*] Pre-intervention to post-intervention changes or changes over time serve as the proxy for the intervention effect in single-arm studies**.**

[†] Other nonprofessionals include nonclinicians such as CHWs, lay workers, volunteers**.**

Abbreviations: BMI=body mass index; CHW=community health worker; CI=confidence interval; ED=emergency department; GEE=generalized estimating equation; HbA1c=hemoglobin A1c, ; MCS=Mental Composite Score; N=number; NR=not reported; NS=not significant; OR=odds ratio; PCS=Physical Component Score; RCT=randomized controlled trial; SE=standard error; SF-12=Short Form-12; SNAP=Supplemental Nutrition Assistance; WIC=Women, Infants, and Children.

© 2023 Cené CW et al. *JAMA Network Open.*

eReferences

1. Siu AL, Bibbins-Domingo K, Grossman DC, et al. Screening for depression in adults: US Preventive Services Task Force recommendation statement. *JAMA*. 2016;315(4):380-7. doi:10.1001/jama.2015.18392
2. Curry SJ, Krist AH, Owens DK, et al. Screening and behavioral counseling interventions to reduce unhealthy alcohol use in adolescents and adults: US Preventive Services Task Force recommendation statement. *JAMA*. 2018;320(18):1899-1909. doi:10.1001/jama.2018.16789
3. O'Connor EA, Evans CV, Rushkin MC, Redmond N, Lin JS. *Behavioral Counseling Interventions to Promote a Healthy Diet and Physical Activity for Cardiovascular Disease Prevention in Adults with Cardiovascular Risk Factors: Updated Systematic Review for the U.S. Preventive Services Task Force*. 2020.
4. Krist AH, Davidson KW, Mangione CM, et al. Screening for unhealthy drug use: US Preventive Services Task Force recommendation statement. *JAMA*. 2020;323(22):2301-2309. doi:10.1001/jama.2020.8020
5. Patnode CD, Henderson JT, Melnikow J, Coppola EL, Durbin S, Thomas R. *Interventions for Tobacco Cessation in Adults, Including Pregnant Women: An Evidence Update for the U.S. Preventive Services Task Force*. 2021.
6. Feltner C, Wallace I, Berkman N, et al. *Screening for Intimate Partner Violence, Elder Abuse, and Abuse of Vulnerable Adults: An Evidence Review for the U.S. Preventive Services Task Force*. 2018.
7. Viswanathan M, Fraser JG, Pan H, et al. Primary care interventions to prevent child maltreatment: updated evidence report and systematic review for the US Preventive Services Task Force. *JAMA*. 2018;320(20):2129-2140. doi:10.1001/jama.2018.17647
8. Community Services Task Force. *Physical Activity: Built Environment Approaches Combining Transportation System Interventions with Land Use and Environmental Design*. 2016. December. https://www.thecommunityguide.org/sites/default/files/assets/PA-Built-Environments.pdf
9. Viswanathan M, Kennedy S, Eder M, et al. *Social needs interventions to improve health outcomes: review and evidence map*. Patient-Centered Outcomes Research Institute; 2021. Prepared by RTI under Contract No. IDIQ-TO#13-RTI EVIDENCEMAPAMPTESP and Contract No. MSA-MDB-ENG-05-26-2020. August.
10. RoB 2: a revised Cochrane risk-of-bias tool for randomized trials. Cochrane. Published 2020. https://methods.cochrane.org/bias/resources/rob-2-revised-cochrane-risk-bias-tool-randomized-trials
11. Sterne JA, Hernán MA, Reeves BC, et al. ROBINS-I: a tool for assessing risk of bias in non-randomised studies of interventions. *BMJ*. 2016;355:i4919. doi:10.1136/bmj.i4919
12. Berkowitz SA, O'Neill J, Sayer E, et al. Health center-based community-supported agriculture: an RCT. Randomized Controlled Trial *Am J Prev Med*. 2019;57(6 Suppl 1):S55-S64. doi:10.1016/j.amepre.2019.07.015
13. Birkhead GS, LeBaron CW, Parsons P, et al. The immunization of children enrolled in the Special Supplemental Food Program for Women, Infants, and Children (WIC): the impact of different strategies. *JAMA*. 1995;274(4):312-6. doi:10.1001/jama.1995.03530040040038
14. Hilgeman MM, Mahaney-Price AF, Stanton MP, et al. Alabama Veterans Rural Health Initiative: a pilot study of enhanced community outreach in rural areas. *J Rural Health*. 2014;30(2):153-63. doi:10.1111/jrh.12054
15. Horwitz SM, Busch SH, Balestracci KM, Ellingson KD, Rawlings J. Intensive intervention improves primary care follow-up for uninsured emergency department patients. *Acad Emerg Med*. 2005;12(7):647-52. doi:10.1197/j.aem.2005.02.015

© 2023 Cené CW et al. *JAMA Network Open.*

16. Kelley L, Capp R, Carmona JF, et al. Patient navigation to reduce emergency department (ED) utilization among Medicaid insured, frequent ED users: a randomized controlled trial. *J Emerg Med*. 2020;58(6):967-977. doi:10.1016/j.jemermed.2019.12.001
17. Krieger J, Collier C, Song L, Martin D. Linking community-based blood pressure measurement to clinical care: a randomized controlled trial of outreach and tracking by community health workers. *Am J Public Health*. 1999;89(6):856-61. doi:10.2105/ajph.89.6.856
18. Krieger J, Takaro TK, Song L, Beaudet N, Edwards K. A randomized controlled trial of asthma self-management support comparing clinic-based nurses and in-home community health workers: the Seattle-King County Healthy Homes II Project. *Arch Pediatr Adolesc Med*. 2009;163(2):141-9. doi:10.1001/archpediatrics.2008.532
19. Krieger J, Song L, Philby M. Community health worker home visits for adults with uncontrolled asthma: the HomeBASE Trial randomized clinical trial. *JAMA Intern Med*. 2015;175(1):109-17. doi:10.1001/jamainternmed.2014.6353
20. Liss DT, Ackermann RT, Cooper A, et al. Effects of a transitional care practice for a vulnerable population: a pragmatic, randomized comparative effectiveness trial. *J Gen Intern Med*. 2019;Epub ahead of printdoi:10.1007/s11606-019-05078-4
21. Melnikow J, Paliescheskey M, Stewart GK. Effect of a transportation incentive on compliance with the first prenatal appointment: a randomized trial. *Obstet Gynecol*. 1997;89(6):1023-7. doi:10.1016/s0029-7844(97)00147-6
22. Nyamathi A, Flaskerud JH, Leake B, Dixon EL, Lu A. Evaluating the impact of peer, nurse case-managed, and standard HIV risk-reduction programs on psychosocial and health-promoting behavioral outcomes among homeless women. *Res Nurs Health*. 2001;24(5):410-22. doi:10.1002/nur.1041
23. Tomita A, Herman DB. The impact of critical time intervention in reducing psychiatric rehospitalization after hospital discharge. *Psychiatr Serv*. 2012;63(9):935-7. doi:10.1176/appi.ps.201100468
24. Towfighi A, Cheng EM, Ayala-Rivera M, et al. Effect of a coordinated community and chronic care model team intervention vs usual care on systolic blood pressure in patients with stroke or transient ischemic attack: the SUCCEED Randomized Clinical Trial. *JAMA Netw Open*. 2021;4(2):e2036227. doi:10.1001/jamanetworkopen.2020.36227
25. Chaiyachati KH, Hubbard RA, Yeager A, et al. Rideshare-based medical transportation for Medicaid patients and primary care show rates: a difference-in-difference analysis of a pilot program. *J Gen Intern Med*. 2018;33(6):863-868. doi:10.1007/s11606-018-4306-0
26. Chaiyachati KH, Hubbard RA, Yeager A, et al. Association of rideshare-based transportation services and missed primary care appointments: a clinical trial. *JAMA Intern Med*. 2018;178(3):383-389. doi:10.1001/jamainternmed.2017.8336
27. Ciaranello AL, Molitor F, Leamon M, et al. Providing health care services to the formerly homeless: a quasi-experimental evaluation. *J Health Care Poor Underserved*. 2006;17(2):441-61. doi:10.1353/hpu.2006.0056
28. Duru OK, Harwood J, Moin T, et al. Evaluation of a national care coordination program to reduce utilization among high-cost, high-need Medicaid beneficiaries with diabetes. *Med Care*. 2020;58 Suppl 6 Suppl 1:S14-S21. doi:10.1097/MLR.0000000000001315
29. Foster SD, Hart K, Lindsell CJ, Miller CN, Lyons MS. Impact of a low intensity and broadly inclusive ED care coordination intervention on linkage to primary care and ED utilization. *Am J Emerg Med*. 2018;36(12):2219-2224. doi:10.1016/j.ajem.2018.04.005
30. Gusmano MK, Rodwin VG, Weisz D. Medicare beneficiaries living in housing with supportive services experienced lower hospital use than others. *Health Aff (Millwood)*. 2018;37(10):1562-1569. doi:10.1377/hlthaff.2018.0070
31. Lindau ST, Makelarski JA, Abramsohn EM, et al. CommunityRx: a real-world controlled clinical trial of a scalable, low-intensity community resource referral intervention. Randomized Controlled

© 2023 Cené CW et al. *JAMA Network Open.*

Trial Research Support, N.I.H., Extramural Research Support, U.S. Gov't, P.H.S. *Am J Public Health*. 2019;109(4):600-606. doi:10.2105/AJPH.2018.304905

32. Mendelsohn AL, Mogilner LN, Dreyer BP, et al. The impact of a clinic-based literacy intervention on language development in inner-city preschool children. *Pediatrics*. 2001;107(1):130-4.
33. Morales ME, Epstein MH, Marable DE, Oo SA, Berkowitz SA. Food insecurity and cardiovascular health in pregnant women: results from the food for families program, Chelsea, Massachusetts, 2013-2015. *Prev Chronic Dis*. 2016;13:E152. doi:10.5888/pcd13.160212
34. Tessaro I, Campbell M, O'Meara C, et al. State health department and university evaluation of North Carolina's Maternal Outreach Worker Program. *Am J Prev Med*. 1997;13(supplement):38-44.
35. Tsai J, Rosenheck RA. Outcomes of a group intensive peer-support model of case management for supported housing. *Psychiatr Serv*. 2012;63(12):1186-94. doi:10.1176/appi.ps.201200100
36. Krieger JW, Takaro TK, Song L, Weaver M. The Seattle-King County Healthy Homes Project: a randomized, controlled trial of a community health worker intervention to decrease exposure to indoor asthma triggers. *Am J Public Health*. 2005;95(4):652-9. doi:10.2105/ajph.2004.042994
37. Szilagyi PG, Schaffer S, Shone L, et al. Reducing geographic, racial, and ethnic disparities in childhood immunization rates by using reminder/recall interventions in urban primary care practices. *Pediatrics*. 2002;110(5):e58. doi:10.1542/peds.110.5.e58
38. Chan TC, Killeen JP, Castillo EM, et al. Impact of an internet-based emergency department appointment system to access primary care at safety net community clinics. *Ann Emerg Med*. 2009;54(2):279-84. doi:10.1016/j.annemergmed.2008.10.030
39. Duncan PW, Bushnell CD, Jones SB, et al. Randomized pragmatic trial of stroke transitional care: the COMPASS Study. Multicenter Study Pragmatic Clinical Trial *Circ Cardiovasc Qual Outcomes*. 2020;13(6):e006285. doi:10.1161/CIRCOUTCOMES.119.006285
40. Glendenning-Napoli A, Dowling B, Pulvino J, Baillargeon G, Raimer BG. Community-based case management for uninsured patients with chronic diseases: effects on acute care utilization and costs. *Prof Case Manag*. 2012;17(6):267-75. doi:10.1097/NCM.0b013e3182687f2b
41. Juillard C, Cooperman L, Allen I, et al. A decade of hospital-based violence intervention: benefits and shortcomings. *J Trauma Acute Care Surg*. 2016;81(6):1156-1161. doi:10.1097/ta.0000000000001261
42. Lapham SC, Hall M, Skipper BJ. Homelessness and substance use among alcohol abusers following participation in project H&ART. *J Addict Dis*. 1995;14(4):41-55. doi:10.1300/j069v14n04_03
43. Lyles CR, Sarkar U, Patel U, et al. Real-world insights from launching remote peer-to-peer mentoring in a safety net healthcare delivery setting. Research Support, Non-U.S. Gov't. *J Am Med Inform Assoc*. 2021;28(2):365-370. doi:10.1093/jamia/ocaa251
44. Martinez TE, Burt MR. Impact of permanent supportive housing on the use of acute care health services by homeless adults. *Psychiatr Serv*. 2006;57(7):992-9. doi:10.1176/ps.2006.57.7.992
45. Slesnick N, Kang MJ, Bonomi AE, Prestopnik JL. Six- and twelve-month outcomes among homeless youth accessing therapy and case management services through an urban drop-in center. *Health Serv Res*. 2008;43(1 Pt 1):211-29. doi:10.1111/j.1475-6773.2007.00755.x
46. Whorms DS, Narayan AK, Pourvaziri A, et al. Analysis of the effects of a patient-centered rideshare program on missed appointments and timeliness for MRI appointments at an academic medical center. *J Am Coll Radiol*. 2021;18(2):240-247. doi:10.1016/j.jacr.2020.05.037
47. Xiang X, Zuverink A, Rosenberg W, Mahmoudi E. Social work-based transitional care intervention for super utilizers of medical care: a retrospective analysis of the bridge model for super utilizers. Research Support, Non-U.S. Gov't. *Soc Work Health Care*. 2019;58(1):126-141. doi:10.1080/00981389.2018.1547345
48. Crisanti AS, Duran D, Greene RN, Reno J, Luna-Anderson C, Altschul DB. A longitudinal analysis of peer-delivered permanent supportive housing: impact of housing on mental and overall health in an ethnically diverse population. *Psychol Serv*. 2017;14(2):141-153. doi:10.1037/ser0000135

© 2023 Cené CW et al. *JAMA Network Open.*

49. Berkowitz SA, Hulberg AC, Standish S, Reznor G, Atlas SJ. Addressing unmet basic resource needs as part of chronic cardiometabolic disease management. *JAMA Intern Med*. 2017;177(2):244-252. doi:10.1001/jamainternmed.2016.7691
50. Gottlieb LM, Adler NE, Wing H, et al. Effects of in-person assistance vs personalized written resources about social services on household social risks and child and caregiver health: a randomized clinical trial. *JAMA Netw Open*. 2020;3(3):e200701. doi:10.1001/jamanetworkopen.2020.0701
51. Guevara JP, Erkoboni D, Gerdes M, et al. Effects of early literacy promotion on child language development and home reading environment: a randomized controlled trial. *J Pediatr X*. 2020;2:100020. doi:10.1016/j.ympdx.2020.100020
52. Izumi BT, Martin A, Garvin T, et al. CSA Partnerships for Health: outcome evaluation results from a subsidized community-supported agriculture program to connect safety-net clinic patients with farms to improve dietary behaviors, food security, and overall health. Research Support, Non-U.S. Gov't. *Transl Behav Med*. 2020;10(6):1277-1285. doi:10.1093/tbm/ibaa041
53. Moreno G, Mangione CM, Tseng CH, et al. Connecting Provider to home: a home-based social intervention program for older adults. Research Support, N.I.H., Extramural Research Support, Non-U.S. Gov't. *J Am Geriatr Soc*. 2021;69(6):1627-1637. doi:10.1111/jgs.17071
54. Seligman HK, Lyles C, Marshall MB, et al. A pilot food bank intervention featuring diabetes-appropriate food improved glycemic control among clients in three states. *Health Aff (Millwood)*. 2015;34(11):1956-63. doi:10.1377/hlthaff.2015.0641
55. Shah R, Chen C, O'Rourke S, Lee M, Mohanty SA, Abraham J. Evaluation of care management for the uninsured. *Med Care*. 2011;49(2):166-71. doi:10.1097/MLR.0b013e3182028e81

© 2023 Cené CW et al. *JAMA Network Open.*
