# Supplementary material for: Racial Health Equity and Social Needs Interventions: A Review of a Scoping Review
Source: JAMA Netw Open. 2023 Jan 19;6(1):e2250654. doi: 10.1001/jamanetworkopen.2022.50654 (PMC9857687; doi:10.1001/jamanetworkopen.2022.50654)
Supplement: Supplement 2. — Data Sharing Statement [file jamanetwopen-e2250654-s002.pdf]

Data Sharing Statement

Cené. Racial Health Equity and Social Needs Interventions. *JAMA Netw Open*. Published January 19, 2023. doi:10.1001/jamanetworkopen.2022.50654

Data
Data available: No
